# Human Antimicrobial RNases Inhibit Intracellular Bacterial Growth and Induce Autophagy in Mycobacteria-Infected Macrophages

**DOI:** 10.3389/fimmu.2019.01500

**Published:** 2019-07-02

**Authors:** Lu Lu, Javier Arranz-Trullén, Guillem Prats-Ejarque, David Pulido, Sanjib Bhakta, Ester Boix

**Affiliations:** ^1^Department of Biochemistry and Molecular Biology, Faculty of Biosciences, Universitat Autònoma de Barcelona, Cerdanyola del Vallès, Spain; ^2^Mycobacteria Research Laboratory, Department of Biological Sciences, Institute of Structural and Molecular Biology, Birkbeck, University of London, London, United Kingdom

**Keywords:** antimicrobial peptides, ribonucleases, tuberculosis, macrophage, autophagy

## Abstract

The development of novel treatment against tuberculosis is a priority global health challenge. Antimicrobial proteins and peptides offer a multifaceted mechanism suitable to fight bacterial resistance. Within the RNaseA superfamily there is a group of highly cationic proteins secreted by innate immune cells with anti-infective and immune-regulatory properties. In this work, we have tested the human canonical members of the RNase family using a spot-culture growth inhibition assay based mycobacteria-infected macrophage model for evaluating their anti-tubercular properties. Out of the seven tested recombinant human RNases, we have identified two members, RNase3 and RNase6, which were highly effective against *Mycobacterium aurum* extra- and intracellularly and induced an autophagy process. We observed the proteins internalization within macrophages and their capacity to eradicate the intracellular mycobacterial infection at a low micro-molar range. Contribution of the enzymatic activity was discarded by site-directed mutagenesis at the RNase catalytic site. The protein induction of autophagy was analyzed by RT-qPCR, western blot, immunofluorescence, and electron microscopy. Specific blockage of auto-phagosome formation and maturation reduced the protein's ability to eradicate the infection. In addition, we found that the *M. aurum* infection of human THP1 macrophages modulates the expression of endogenous RNase3 and RNase6, suggesting a function *in vivo*. Overall, our data anticipate a biological role for human antimicrobial RNases in host response to mycobacterial infections and set the basis for the design of novel anti-tubercular drugs.

## Introduction

Tuberculosis (TB) is an ancient life-threatening infectious disease currently rated among the top ten causes of death worldwide. According to the World Health Organization (WHO), TB is responsible for about 1.6 million TB deaths and 10 million (5.8 million men, 3.2 million women, and 1.0 million children) new cases have been detected in 2017 and more than a third of the world population is hosting *Mycobacterium tuberculosis*, the causative pathogen of TB, in its latent form (1, 2). In recent years, we have seen a general decline in the TB incidence rate as well as in the absolute number of registered cases of tuberculosis. Nonetheless, the emergence of a growing number of new cases of multi-drug resistant TB (MDR-TB) and even more alarming extensively drug-resistant TB (XDR-TB) cases have placed the eradication of TB as one of the major global challenges to overcome in the twenty-first century. Furthermore, TB prognosis is significantly aggravated during human immunodeficiency virus (HIV) co-infection. A complex cellular machinery is activated during the host immune response against *M. tuberculosis* (Mtb) bacilli; unfortunately, their underlying mechanisms remain poorly understood. Hence, enlargement of novel and effective therapies to battle mycobacteria are urgently required (3, 4). Mtb is an intracellular pathogen able to survive indefinitely under unfavorable conditions inside primary host immune cells, preferably residing in human alveolar macrophages (5, 6). Several studies have recently shown that features such as its high infectivity, slow growth, and complex cell-wall structure, make *M. tuberculosis* a major challenge to be faced, since the specific mechanisms that reinforce the high virulence of Mtb remain largely unknown (4, 7). Despite the antimicrobial activity of macrophages, Mtb has been able to establish a series of strategies to handle the host immune machinery, interfere with, and arrest the phagosome maturation, counteract mycobactericidal molecules and ultimately survive in a hostile intracellular environment (8–10). Therefore, the search for new anti-TB agents that are able to safely penetrate the host immune cells and eradicate the pathogen intracellularly is now one of the priorities of global health action programs to tackle tuberculosis.

The eventual outcome of a mycobacterial infection largely depends on the readiness of the host immune system to counter the pathogen. During infection a large assortment of antimicrobial protein and peptides (AMPs) are released by host immune cells to the bloodstream and nearby tissues to the infected areas (11, 12). At the same time, neutrophil, and eosinophil granules, loaded with lytic enzymes, and antimicrobial molecules, can be acquired by macrophages to counter bacterial invasion (13–15). Because AMPs exert a potent anti-infective effect against a wide range of human pathogens and the likelihood of microbial resistance is mostly reduced in comparison to conventional antibiotics, they are emerging as a new generation of natural lead candidates and its administration in combination with or without other drugs is showing highly promising results (16).

Our research group works on the mechanism of action of human antimicrobial RNases, a group of proteins that belong to the RNase-A superfamily. The family encompasses eight functional members in humans, referred as the “canonical RNases” (17). Among them, there are proteins secreted by epithelial and blood cell types during host-defense response that are endowed with the characteristic biophysical features of AMPs (12, 18, 19). In particular, human RNase3, RNase6, and RNase7 are highly cationic proteins with reported antimicrobial activity against a variety of microbes (20–23). Interestingly, secretion of RNases 3 and 7 was associated to *Mycobacterium bovis* (*M. bovis)* BCG and *M. tuberculosis* infection, respectively (14, 24). Indeed, both RNases displayed antimycobacterial activity *in vitro* (25).

In this work, we aimed to study the antimycobacterial activity of the human canonical RNases by applying an integrated surrogate model for screening of anti-tubercular drugs against *M. tuberculosis* (8). The semi-solid agar-based spot-culture growth inhibition assay (SPOTi) has facilitated a rapid high-through screening of antimicrobial agents against *Mycobacterium aurum* (*M. aurum*) using a macrophage infected model (8, 26, 27). *M. aurum* was chosen as a relatively fast-growing and stable intracellular dwelling species within infected macrophages, previously validated as an appropriate surrogate model for Mtb (8). RNases with positive antimycobacterial activity were further characterized by exploring their mechanism of action in macrophages. The present results indicate that the protein antimycobacterial action is partly mediated by the induction of an autophagy process.

## Materials and Methods

### Protein Expression and Purification

The cDNA for RNase1 was a gift from Prof. Maria Vilanova (Universitat de Girona, Spain) and RNase5 from Prof. Demetres Leonidas (University of Thessaly, Greece). RNase4 synthetic gene was purchased from Nzytech company and RNase6 was obtained from DNA 2.0 (Menlo Park, CA, USA). RNase2, RNase3, and RNase7 genes were obtained as previously described (20). Active site mutations into the RNases genes were introduced using the Quick change™ site-directed mutagenesis kit (Agilent, 200523) following the manufacturers procedure (28). *Escherichia coli (E. coli)* BL21 (DE3) (Novagen, 69450) competent cells were transformed with the pET11c/RNase plasmids. Recombinant proteins were expressed and purified in *E. coli* BL21 (DE3) by an adaptation of the protocol previously described (20). Briefly, *E. coli* BL21(DE3) cells were induced by 1 mM isopropyl β-D-1-thiogalactopyranoside (G Bioscience, RC-063) and the inclusion bodies enriched pellet was resuspended in 80 mL of 10 mM Tris-HCl pH 8.5, 2 mM EDTA and left incubating 30 min with 40 μg/mL of lysozyme prior to sonication. Then, the sample was centrifuged at 30.000 × *g* for 30 min at 4°C and the pellet was resuspended in 25 mL of the same buffer supplemented with 1% triton X-100 and 1 M urea and was left stirring at room temperature for 30 min and centrifuged 30 min at 22.000 × *g*. Following, 200 mL of 10 mM Tris-HCl pH 8.5, 2 mM EDTA was added to the pellet, and then the sample was centrifuged at 22.000 × *g* for 30 min (4°C). The resulting pellet solubilized in 6 M guanidine hydrochloride and rapidly 80-fold diluted in the refolding buffer was left in gentle stirring for 48–72 h at 4°C. The folded protein was then concentrated, dialyzed against the chromatography buffer and purified by cation exchange chromatography on a Resource S column (GE Healthcare Life Sciences, GE17118001). The identity and homogeneity of the purified proteins were checked by SDS-PAGE, MALDI-TOF, and N-terminal sequencing.

### Macrophage Cell Culture

Mouse RAW 264.7 cells (NCTC, #91062702) and human THP-1 cells (NCTC, #88081201) were maintained or passaged in 25 cm^2^ tissue culture flasks (BD Biosciences, 353108) using DMEM (Lonza, BE04-687F/U1) and RPMI-1640 (Lonza, BE12-702F) medium with 10% heat-inactivated fetal bovine serum (FBS, Gibco, 26140079) respectively at 37°C and humidified 5% CO_2_ conditions. RAW 264.7 cells were seeded at 5 × 10^5^ per well and allowed to attach for 2 h before infection and treatment. THP-1 cells were treated with 100 nM phorbol myristate acetate (PMA, Sigma-Aldrich, P8139) for 48 h to induce differentiation into macrophage-like cells and allowed to rest for 24 h before further treatment. The number of viable cells was counted using a Trypan blue (Invitrogen, 15250-061) exclusion assay.

### Growth of Mycobacteria Culture and Macrophage Infection

*Mycobacterium aurum* was purchased from the UK National Collection of Type Cultures (NCTC). Cells cultures of *M. aurum* (NCTC, 10437), *Mycobacterium smegmatis* mc^2^155 (ATCC, 700084) and *M. bovis* BCG Pasteur (ATCC, 35734) were grown in Middlebrook (MB) 7H9 broth (BD Biosciences, 271310) enriched with 10% (v/v) albumin/dextrose/catalase (ADC; BD Biosciences, 212352) containing 0.05% Tween 80 and 0.05% Glycerol for liquid growth at 37°C for BCG, and in MB7H10 (BD Biosciences, 262710) with 10% (v/v) oleic acid/albumin/dextrose/catalase (OADC; BD Biosciences, 212240) for semi-solid agar growth at 37°C. Stock cultures of log-phase cells were maintained in glycerol (25% final concentration of glycerol) at −80°C. The bacteria were vortexed and sonicated using ultrasound sonication bath to obtain a single cell suspension, and then the bacterial concentration was determined by measuring the optical density (OD) of the culture at 600 nm (1 OD = 10^9^ CFU/mL). Mid-log phase *M. aurum* cells, harvested in RPMI-1640 complete medium, were co-cultured with RAW264.7 or THP-1 at a multiplicity of infection (MOI) of 10:1 and were incubated at 37°C for 3 h, then were washed 3 times with PBS and replaced with fresh media supplied with 50 μg/mL gentamycin (Apollo Scientific, BIG0124) to remove extracellular mycobacteria during further treatment.

### High Throughput SPOTi Assay, Minimum Inhibitory Concentration (MIC), and Minimum Bactericidal Concentration (MBC) Assays

Antimicrobial activity of RNases was evaluated by calculating the 100% minimum bactericidal concentration (MBC_100_) and 100% minimum inhibitory concentration (MIC_100_) in mycobacteria cultures. The semi-solid agar-based SPOTi assay system was applied for fast and high throughput screening and the colony forming unit (CFU) counting assay for the validation and accurate MBC/MIC_100_ determination (8). The MBC_100_ and MIC_100_ of each protein was determined from two independent experiments performed in triplicate for each concentration. Briefly, log-phase cultures of mycobacteria (OD_600_≈1) were first checked for quality control using cold Ziehl–Neelsen (ZN) staining (also called “acid fast staining”; TB-color staining kit, Merck Millipore, 116450) according to the manufacturer's protocol. For extracellular analysis, the mycobacterial cultures were then diluted to 2 × 10^5^ CFUs/mL and directly incubated with proteins at 37°C for 4 h in PBS in 1 mL. For intracellular analysis, the mycobacterial cultures diluted to 2 × 10^5^ CFUs/mL were used to infect macrophages and then treated with the proteins for up to 72 h in RPMI-1640 medium with 10% FBS. MIC_100_ were calculated at final incubation time. Uptake of mycobacteria by RAW macrophages was confirmed using the ZN staining assay, as described (8). No significant reduction in the number of live macrophages was observed during the experiment. Macrophage infection was monitored by CFUs counting. Following the treatment, to evaluate the activity at the protein extracellular activity the cultures were centrifuged and suspended in 50 μL of distilled water and 5 μL of the sample was spotted onto wells of a 24-well plate containing MB7H10/OADC/agar. For evaluation of the protein intracellular activity, the macrophages were washed twice with RPMI-1640 and lysed in water to harvest intracellular mycobacteria for plating in 24-well plate (SPOTi) or 10 mm petri dish (CFU assay). Initial infection was adjusted to 2 × 10^5^ CFU/mL for all samples. Mycobacterial cell growth at 37°C was recorded after 2 days for *M. smegmatis*, 4–5 days for *M. aurum*, and 14 days for *M. bovis* BCG.

### Minimum Agglutination Activity (MAC) Assay

Mycobacterial cells were grown at 37°C to an OD600 of 1.0 and diluted ten times. Then, cells were centrifuged at 5,000 × g for 2 min, and resuspended in M7H9/ADC media containing 0.05% Tween-80 and 0.05% glycerol. An aliquot of 100 μl of the mycobacterial suspension was treated with increasing protein concentrations (from 0.01 to 25 μM) and incubated at 37°C for 1 h. The aggregation behavior was observed by visual inspection with a stereomicroscope at 50x, and the agglutinating activity was calculated as the minimum agglutinating concentration of the protein, as previously described Pulido et al. (22).

### Cytotoxicity Assay

To assay the toxicity of RNases to RAW264.7 macrophages, the assay was performed in 96-well cell culture flat-bottom plates (Costar; Appleton Woods) in triplicate. To each well, 100 μL of diluted macrophage cells (5 × 10^5^ cells/mL) were dispensed in 96-well plates and then proteins were added serially at final concentrations ranging from 0.01 to 50 μM (100 μL/well) and incubated for 48 h; cells were then washed twice with 1X PBS, and fresh RPMI-1640 complete medium was added. Plates were then treated with 30 μL of a freshly prepared 0.01% resazurin solution and incubated overnight at 37°C. The following day the change in color was observed and the fluorescence intensity was measured (λ_em_560, λ_ex_590 nm, FLUOstar OPTIMA microplate reader; BMG LABTECH GmbH). The 50% growth inhibitory concentration (IC_50_) was determined.

### Tracking of RNases Internalization Into RAW264.7 Macrophages by Confocal Microscopy

RNase3, RNase6, and RNase7 were labeled with Alexa Fluor 488 Labeling kit (Invitrogen, A10235), following the manufacturer′s instruction as previously described (21). To 0.5 mL of 2 mg/mL protein solution in PBS, 50 μL of 1 M sodium bicarbonate, pH 8.3, was added. The protein was incubated for 1 h at room temperature with the reactive dye, with stirring, and the labeled protein was separated from the free dye by PD-10 desalting column (GE Healthcare, 17-0851-01). Labeled protein distribution in cell cultures was followed by confocal microscopy. About 2.5 × 10^5^ RAW cells were harvested in 3 cm diameter microscopy plates (MatTek, P35G-1.5-14-C) 2–3 h before the assay. Macrophages were washed with RPMI and labeled with Hoescht 33342 (Thermo Fisher Scientific, 62249) and Cell Mask Deep Red Plasma membrane Stain (Thermo Fisher Scientific, C10046) at 0.5 μg/mL for 5–10 min before observation in Leica TCS SP5 AOBS equipped with a PL APO 63 × 1.4–0.6 CS oil immersion objective (Leica Microsystems, Mannheim, Germany). Following, Alexa Fluor labeled proteins were added at 2 μM to the cultures and time lapse was recorded at intervals of 30 s for 30 min. Fluorochromes were excited by 405 nm (Hoechst 33342), 649 nm (CellMask Deep Red), and 488 nm (Alexa Fluor 488). Emissions were collected with a HyD detector.

### Real-Time qPCR Assays

Total RNA was extracted using mirVanaTM miRNA Isolation Kit as described by the manufacturer (Ambion, Life Technologies, AM1560) at each time point (4, 24, 48, 72 h). Total RNA was quantified by NanoDropTM spectrophotometer (Thermo Fisher Scientific, Wilmington, DE USA), and cDNA was synthesized using iScriptTM cDNA Synthesis Kit (Bio-Rad, 170-8891). The unique ID of the primers used (synthesized by Bio-Rad, Hercules, CA, USA) is shown in Table S1. Transcripts of human *RNase2, RNase3, RNase6*, and *RNase7* genes relative to the human housekeeping *GAPDH*, and mouse *BECN1* and *ATG5* genes relative to mouse housekeeping β*-actin* were measured in triplicate from cDNA samples by real-time quantitative PCR using CFX96 Real-Time PCR detection system (Bio-Rad, Hercules, CA, USA). The results were analyzed by using the relative standard method (29).

### Western-Blot Analysis

Expression level of the autophagy marker LC3 in RAW264.7 macrophages was evaluated by western blot analysis. Autophagy was inhibited by addition of either 100 nM of Bafilomycin A (BA, Sigma-Aldrich, 19-148) or 5 mM of 3 Methyladenine (3MA, Sigma-Aldrich, M9281) (30, 31). Rapamycin (Sigma-Aldrich, R0395) at 100 nM was used as a positive control. When the treatment was finished, cells were lysed by RIPA buffer and the protein concentration was determined with the Pierce BCA Protein Assay kit (Thermo Fisher Scientific, 23225). Equal amounts of protein (50 μg) for each sample were loaded onto a 15% SDS–PAGE gel. After the electrophoresis, proteins were transferred to PVDF membrane. The membranes were blocked and incubated with the primary antibody of interest. The primary antibodies used were rabbit polyclonal anti-LC3 (1:1,000, Abcam, ab48394) and chicken polyclonal anti-GAPDH (1:2,000, Abcam, ab9483). After washing, a horseradish peroxidase-conjugated antibody (Goat Anti-Rabbit IgG antibody, Sigma Aldrich, 12-348 and Goat anti-chicken IgY H&L, Abcam, ab6877) was applied for detection using an enhanced chemiluminescent detection system (Supersignal West Pico Chemiluminescent Substrate, ThermoFisher Scientific, 32209). Densitometry analysis was performed using Quantity One software.

### Immunofluorescence Microscopy

RAW264.7 cells were grown, plated, and infected as above on glass coverslip (5 × 10^5^ cells each one). After fixation and blocking, cells were incubated with the primary antibodies anti-LC3 (1:200, Abcam, ab48394); the cultures were incubated for 1 h at room temperature with labeled secondary antibodies (1: 500, anti-rabbit IgG-Alexa Fluor 488, ThermoFisher Scientific, A-11008) and then washed 3 times with PBS. Cells were incubated with Hoechst (ThermoFisher Scientific, 62249) and Cell Mask Deep Red (Invitrogen, C10046) to visualize the nuclei and membrane, and then were loaded with mounting media and sealed with nail oil. Images were captured by Leica TCS SP5 AOBS microscope equipped with a PL APO 63 × 1.4–0.6 CS oil immersion objective (Leica Microsystems, Mannheim, Germany). Quantification of fluorescence intensity was performed by using *Imaris* software.

Alternatively, to quantify LC3 accumulation RAW264.7 cells were plated on glass coverslip (5 × 10^5^ cells each one) and treated with RNase3 and/or Bafilomycin A. After treatment, cells were fixed with 2% paraformaldehyde, permeabilized with 0.05% triton-X100, and blocked with 5% non-fat milk. Cells were incubated with the primary antibodies anti-LC3 at 4°C overnight (1:200, Abcam, ab48394), and room temperature 2 h with secondary anti-rabbit IgG-Alexa 488 antibody(ThermoFisher Scientific, A-11008) and then washed 3 times with PBS. Cells were stained with DAPI (ThermoFisher Scientific, 62248) to visualize the nuclei for 30 min at room temperature and then were loaded with mounting media and sealed with nail oil. Images were captured by Leica DM RB equipped with a Leica DFC 500 camera under 100-fold oil objective. LC3 puncta vesicle quantification was performed using *Image J*.

### Transmission Electron Microscopy

For transmission electron microscopy (TEM) analysis, 10^6^ infected RAW264.7 macrophages cells were seeded in 6-well tissue culture plates and were treated with 10 μM RNase3 or RNase3-H15A for 24 h. After treatment, macrophages were fixed with 2% (w/v) paraformaldehyde and 2.5% (v/v) glutaraldehyde in PBS for 1 h. The samples were then dehydrated in acetone (50, 70, 90, 95, and 100%) and were immersed in Epon resin (32). The ultrathin sections were examined in a JEOL JEM 2011 instrument (JEOL, Ltd., Tokyo, Japan).

### Acidic Vesicular Compartment Quantification by the Acridine Orange Assay

To quantify the volume/amount of the acidic vesicular compartment, RAW264.7 macrophage cells were treated with the proteins and were stained with acridine orange (AO, ThermoFisher Scientific, A1301) as previously described (33). Briefly, cells were seeded in 96-well plates (10^4^ cells/well) and treated with RNases for 24 h. Rapamycin (100 nM) was used as a positive control and BA (100 nM) was used as an inhibitor of acidification of vesicular content. At the end of the treatments, cells were washed with pre-warmed PBS and stained with 5 μg/mL AO for 10 min in PBS. After washing with PBS three times, the increase in the amount of the acidic vesicular compartment was quantified by measuring the red/green fluorescence intensity ratio of AO staining (AO green fluorescence λ_exc_ 485 and λ_em_ 535 nm; AO red fluorescence λ_exc_ 430 and λ_em_ 590 nm). Values were normalized by cell proliferation count by the Thiazolyl Blue Tetrazolium Bromide (MTT, Sigma-Aldrich, M5655-100MG) assay.

### Liposome Aggregation Assay

Large unilamellar vesicles (LUVs) were prepared as previously described (20). Briefly, 1,2-dioleoyl-sn-glycero-3-phosphocholine (DOPC, Avanti, 850375) and 1,2-dioleoyl-sn-glycero-3-phospho-(1'-rac-glycerol) (DOPG, Avanti, 840445P) were mixed in chloroform with 3:2 molar ratio and used to prepare LUVs by Rotavapor. Then the liposomes were resuspended with 10 mM Tris/HCl, pH7.4 and generated a defined size (100 nm) by extrusion through 100 nm polycarbonate membranes.

Aggregation of liposomes was monitored by recording the scattering intensity with the excitation and emission wavelengths at 470 nm using Cary Eclypse spectrofluorimeter. Liposomes were incubated with proteins for indicated time at room temperature, the signal was read 90 degree from the excitation beam with slits at 2.5 and 5 nm.

### Statistical Analysis

Data are presented as mean ± SD and comparisons between groups were analyzed by paired Student *t*-test and one-way ANOVA for comparing more than 2 groups. *p* < 0.05 was considered as statistically significant.

## Results

### Screening of Human Canonical RNases Identifies Three Members With Antimycobacterial Activity at Extracellular and Intracellular Levels

**Figure 1:**
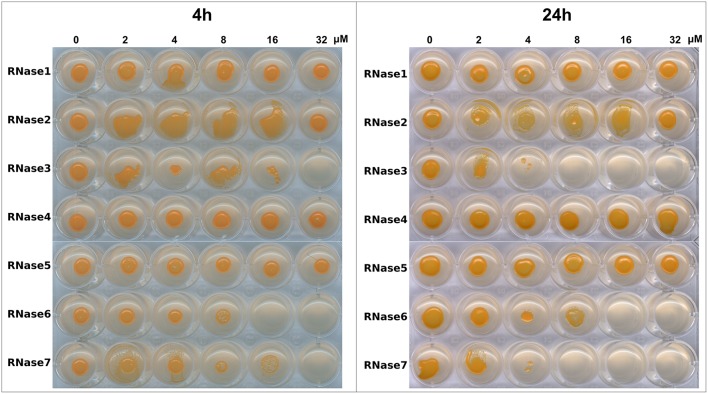
A representative SPOTi image comparing human RNases activity on *Mycobacterium aurum* infected macrophages. RAW 264.7 macrophages were infected with *M. aurum* at 10:1 MOI for 3 h at 37°C. The culture was washed with RPMI-1640 thrice and incubated with different concentrations of the proteins in RPMI-1640 complete medium for 4 h (**Left**) or 24 h (**Right**). Macrophages were washed twice with RPMI-1640 and lysed. Then, an aliquot was spotted onto wells of a 24-well plate containing MB7H10/OADC/agar and incubated at 37°C for 4–5 days to determine intracellular survival.

In this work we have evaluated for the first time the antimicrobial potency of the seven human RNases from the RNase A superfamily using the SPOTi surrogate model adapted for the screening of novel anti-TB drug candidates (8). Previous work in our laboratory selected *M. aurum* as a fast non-pathogenic species suitable for the screening of anti-tubercular drugs at the extracellular (directly on mycobacterial culture) and intracellular (on mycobacteria residing inside infected macrophage cell-line) levels, referred in this article as *in vitro* and *ex vivo* assays, respectively. Screening results indicated that RNase3, RNase6 and RNase7 can completely inhibit the growth of *M. aurum* cultures at a low micromolar concentration range, while the rest of the RNases did not reduce the mycobacteria population even at the highest concentration tested (Figure S1). Next, we assayed the antimicrobial activity of the seven human RNaseA protein family members against *M. aurum* within RAW 264.7 mouse macrophages. Results from the screening of the seven human secreted RNases confirmed that only RNase3, RNase6 and RNase7 are active against mycobacterial infection (Figure 1). Interestingly, when comparing the reduction of CFU counts in comparison to control untreated samples for *in vitro* and *ex vivo* conditions we observed that the effectiveness of the protein was higher at the intracellular level (*ex vivo* assay) (Table 1). Besides, comparison of the macrophage intracellular antimycobacterial activity at 4 and 24 h indicated that the protein activity is enhanced at longer incubation times. Total inhibition of intracellular *M. aurum* growth was achieved at about 2-fold lower protein concentration at 24 h incubation time in comparison to 4 h (Figure 1). Monitoring of the protein activity within infected macrophages up to 72 h also revealed an increase of the antimicrobial efficiency at longer exposure times. The results suggested that other mechanisms, together with a direct killing of the bacilli, are taking place.

**Table 1:** Comparison of the antimycobacterial activity of RNase3, RNase6, and RNase7 and their respective active center mutants (H15A).

**Protein**	**Extracellular MBC (μM)**			**MAC (μM)**			**Intracellular MIC (μM)**	**IC_**50**_ (μM)**
	***M. aurum***	***M. smegmatis* mc^**2**^155**	***M. bovis* BCG**	***M. aurum***	***M. smegmatis* mc^**2**^155**	***M. bovis* BCG**	***M. aurum***	**RAW264.7**
RNase3	18.75 ± 0.05	9.37 ± 0.05	18.75 ± 0.05	2.34 ± 0.5	1.17 ± 0.3	4.68 ± 1.0	5 ± 0.05	> 25
RNase3-H15A	18.75 ± 0.05	18.75 ± 0.05	18.75 ± 0.05	4.68 ± 0.5	4.68 ± 1.0	4.68 ± 1.0	10 ± 0.05	> 25
RNase6	18.75 ± 0.05	18.75 ± 0.05	18.75 ± 0.05	4.68 ± 1.0	2.34 ± 0.5	4.68 ± 1.0	10 ± 0.05	> 25
RNase6-H15A	18.75 ± 0.05	18.75 ± 0.05	37.5 ± 0.05	4.68 ± 1.0	2.34 ± 0.5	4.68 ± 1.0	10 ± 0.05	> 25
RNase7	18.75 ± 0.05	18.75 ± 0.05	18.75 ± 0.05	> 10	> 10	> 25	10 ± 0.05	> 25
RNase7-H15A	18.75 ± 0.05	18.75 ± 0.05	18.75 ± 0.05	> 10	> 10	> 25	10 ± 0.05	> 25

*Extracellular minimum bactericidal concentration (MBC_100_) and intracellular minimum inhibitory concentration (MIC_100_) were calculated at 72 h incubation time using the SPOTi assay. Minimum agglutination concentration (MAC) and Inhibitory Concentration (IC_50_) for RAW 264.7 mouse macrophages were assayed as described in the methodology. Results are an average of three independent repeated experiments. Mean average values ± SD are indicated*.

### The Enzymatic Activity Is Not Involved in the RNases Antimycobacterial Activity

Following, RNase3, RNase6, and RNase7 were chosen to further investigate whether the enzymatic activity contributes to the antimycobacterial properties of human RNases by comparing the recombinant native proteins with their catalytic inactive mutants. Previous work from our laboratory confirmed that the H15A substitution fully removed the catalytic activity without altering the protein 3D-structure (28). First, the three antimicrobial proteins (RNases 3, 6, and 7) together with their respective active site mutants (H15A) were tested *in vitro* against *M. smegmatis* mc^2^155, *M. aurum* and *M. bovi*s BCG. As summarized in Table 1, RNase3, RNase6, and RNase7 showed similar antimicrobial activities against the three mycobacteria species. Then, *M. aurum* species was chosen for macrophage intracellular studies, being among the three tested species the only one that provides a fast growing profile together with an efficient macrophage uptake (8). Overall, no significant differences were observed between the native proteins and their respective active site mutants against *M. aurum*, both at the extracellular and intracellular levels. Therefore, we can conclude that the three RNases can eradicate *M. aurum* dwelling within the macrophage at a low micromolar concentration range. To note, RNase3 stands out as the most active, achieving mycobacterial growth inhibition at 5 μM (Table 1).

Next, we tested the agglutinating activity of the RNases against the three assayed mycobacterial species. The agglutinating activity was calculated as the minimum agglutinating concentration (MAC). Results indicated that RNase3 and RNase6 can agglutinate mycobacteria within the same concentration range, while RNase7 is not active even at the maximum concentration tested. Besides, there were no significant differences among the MAC values of the wild type and active site mutants (Table 1). From our findings, we can infer that catalytic activity of the ribonucleases is not involved in their antimycobacterial mode of action.

### Tracking the Protein Internalization Within Macrophages

**Figure 2:**
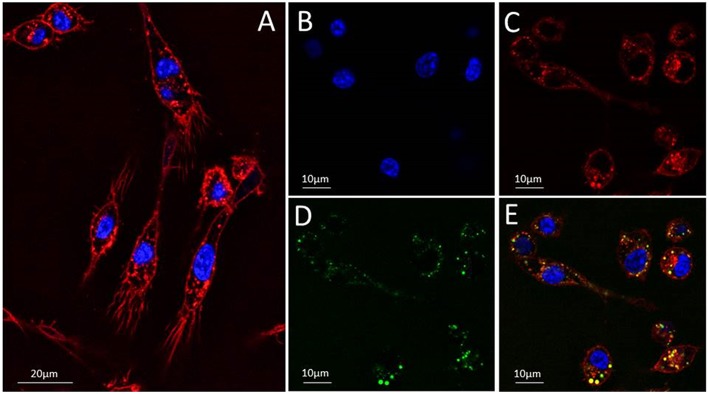
Tracking the translocation of Alexa Fluor 488-labeled RNase3 into RAW 264.7 mouse macrophages. **(A)** macrophages cells were stained with Hoechst and Deep Red to visualize the nuclei (blue) **(B)** and membranes (red) **(C)**, respectively. **(D)** The protein location was registered after 30 min of macrophages post-treatment with Alexa Fluor 488-RNase3 (green). A panel showing merged images is shown in **(E)**. Protein tracking assays were performed using 2.5 × 10^5^ cells/mL and adding 2 μM of labeled protein final concentration. The images were taken using a Leica TCS SP5 AOBS microscope (see Materials and Methods section for more details).

Following, the RNases internalization within macrophages was visualized by confocal microscopy. Results confirmed that the three RNases are able to internalize within the mouse macrophages through a vesicle-mediated mechanism (Figure 2). Analysis of the profiles of selected regions of interest confirmed the protein confined location within macrophage vesicles (Figures S2, S3). In addition, time-lapse assays indicated that internalization is a fast process, where most of the protein enters into the macrophage within the first 30 min. Examples of internalization fluorescence profiles of labeled RNase3, RNase6, and RNase7 were recorded at several time intervals (Figure S4). Confocal microscopy images confirmed that no damage to the macrophage cells at the assayed conditions was taking place. Besides, the protein toxicity on the mouse macrophages in the assayed conditions was also discarded by using the resazurin assay; no significant cytotoxicity was observed at the maximum concentration tested (25 μM) (Table 1).

### *M. aurum* Infection Modulates the Expression of RNases in THP1 Macrophage Differentiated Cells

Encouraged by positive results on the antimycobacterial activity of recombinant RNases we decided to test whether the mycobacteria infection can induce the protein expression in human macrophages. We selected the human monocyte THP1 cell line differentiated to macrophages and infected it with *M. aurum*. Levels of expression of human RNases were evaluated by real time qPCR. Primers of the four known antimicrobial RNases expressed in blood cell type were used to quantify their transcription levels (Table S1). Transcription levels were quantified in relation to the *GAPDH* housekeeping gene. Results indicated that both control macrophage derived THP1 cells and *M. aurum* infected cells expressed the tested RNase genes (Figure S5). However, the protein transcription rates differed significantly among the RNases. Results of non-infected THP1 cells were in agreement with previous expression profile reports showing the predominant transcription of *RNase2* (18). Comparison of the relative expression levels highlighted also the significant quantities of *RNase6* followed by *RNase3*. On the contrary, *RNase7* expression was very scarce, more than 1,000-fold lower than *RNase2*. Interestingly, *M. aurum* infection modulated the expression of *RNase3* and *RNase6*, while *RNase2* transcription levels remained unaffected (Figure S5). In addition, analysis of the expression profiles as a function of time also highlighted significant differences between early and late infection periods. Overall, *M. aurum* infection significantly inhibited the THP1 cells expression of RNases at the beginning of the infection (24 h), but triggered their expression at longer infection periods (48–72 h).

### RNases 3 and 6 Induce Autophagy in Macrophages

**Figure 3:**
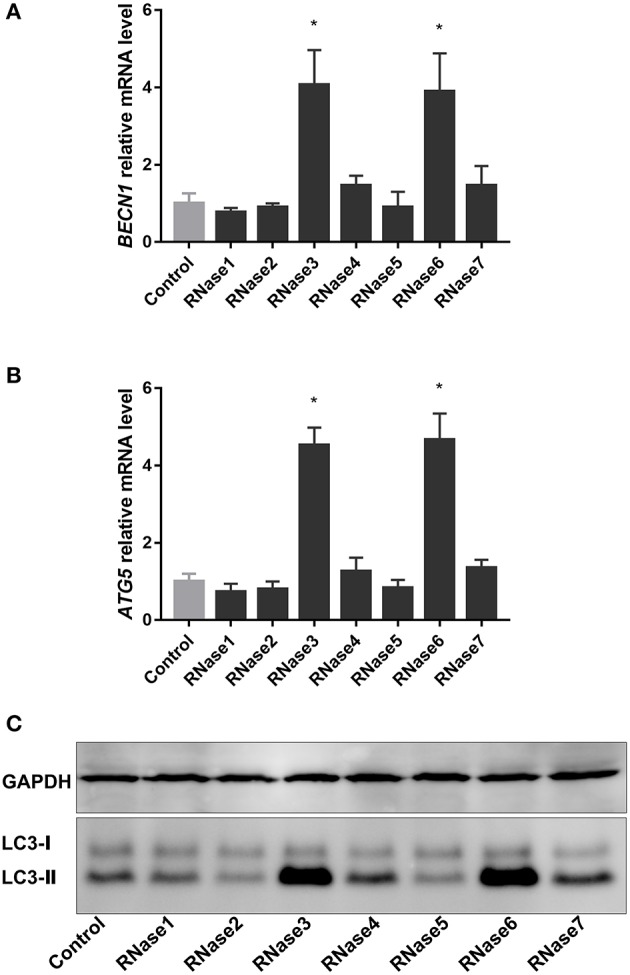
Human RNases 3 and 6 induce macrophage autophagy. RAW 264.7 macrophages were infected with *M. aurum* and treated with 10 μM of proteins (RNase1 to RNase7) for 24 h. Real-time qPCR measured the relative expression of *BECN1* **(A)** and *ATG5* **(B)** genes normalized by housekeeping gene β*-actin*. Results are shown from 3 independent experiments (mean ± SD); **(C)** Immunoblot of LC3-processing and the housekeeping GAPDH control. Results are shown from 3 independent experiments (mean ± SD). *Statistically significant differences compared with the control sample are indicated (*p* value < 0.05).

Autophagy is one of the main mechanisms activated by infected macrophages to remove intracellular resident mycobacteria (34). Considering that some AMPs are known to participate in the host immune response against mycobacteria by the activation of autophagy (12), we decided to explore the action of human RNases on infected macrophages. RAW 264.7 mouse macrophages were chosen as a stable well-characterized cell line for the analysis of the recombinant proteins. First, we tested whether the human RNases could induce the expression of the autophagy markers *BECN1* and *ATG5*, two essential genes involved in autophagosome formation and maturation, respectively. Analysis of qPCR results indicated that RNase3 and RNase6, out of the seven screened RNases, significantly upregulated the mRNA expression of *BECN1* and *ATG5* in RAW 264.7 macrophages (Figures 3A,B). These positive results were corroborated by analysis of LC3 processing and detection of the LC3II form, involved in the autophagosome formation (35). LC3 transformation was also evaluated by immunoblotting, confirming an increase in the LC3II fraction in detriment of LC3I in RNase3 and RNase6 treated samples in comparison to control (Figure 3C). Therefore, we conclude that both RNases activate an autophagy process in the treated macrophages.

### RNase Catalytic Activity Is Not Required for the Protein Autophagy Activation

**Figure 4:**
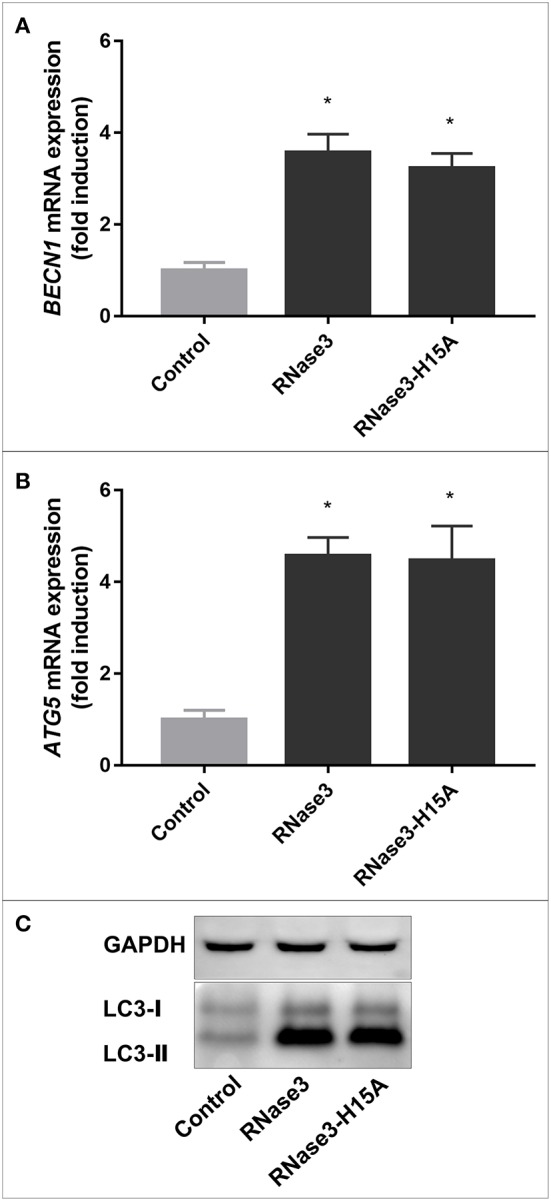
Both RNase3 and the RNase-H15A active site mutant induce autophagy in macrophages. RAW 264.7 mouse macrophages were infected with *M. aurum* and treated with 10 μM of protein (RNase3 and RNase3-H15A). Real-time qPCR measured the relative expression of *BECN1* **(A)** and *ATG5* **(B)** genes normalized by the housekeeping gene β*-actin*. Results are shown from 3 independent experiments (mean ± SD); **(C)** Immunoblot of LC3-processing and the housekeeping GAPDH control. Results are shown from 3 independent experiments (mean ± SD). *Statistically significant differences compared with the control sample are indicated (*p* value < 0.05).

We then analyzed in more details the protein activation of the autophagy pathway by using as a reference RNase3, the most active family member. First, the RNase3 wild-type ability to induce autophagy was compared with the RNase3-H15A enzymatically inactive variant by following the expression of *BECN1* and *ATG5* and monitoring the LC3II/LC3I ratio (Figure 4). Equivalent activity of both wild-type and H15A mutant proteins discards any contribution of the enzyme catalytic activity in the autophagy pathway activation.

**Figure 5:**
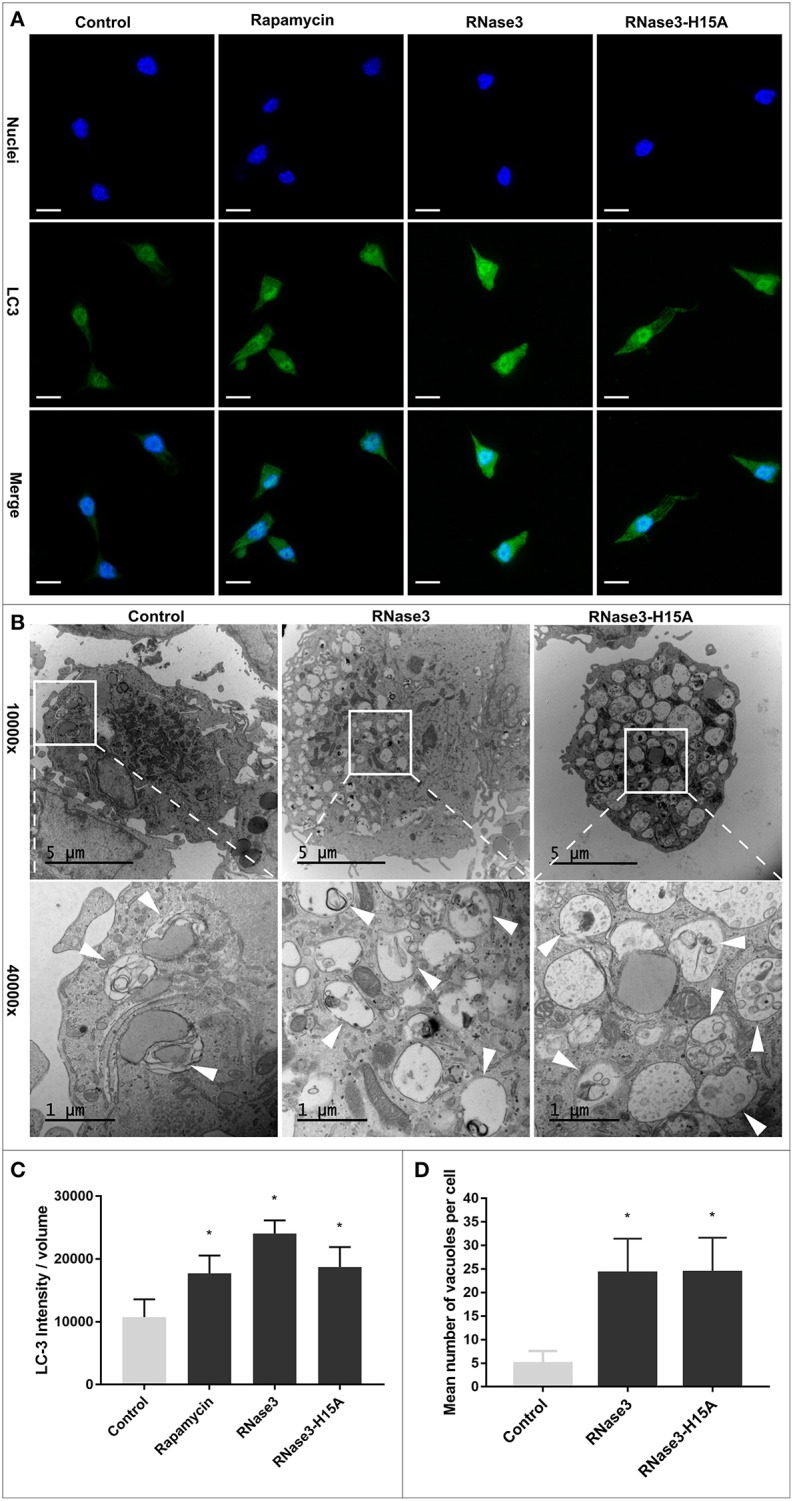
RNase3 and RNase3-H15A induce autophagic vacuole formation and LC3 processing. **(A)** Immunofluorescence microscopy analysis of LC3 processing, Mouse macrophages were infected with *M. aurum* and treated with 10 μM of RNase3, or 10 μM RNase3-H15A mutant, or 100 nM Rapamycin for 24 h. After treatment, the cells were fixed and stained with Hoechst to visualize the nuclei (blue), and with anti-LC3 followed by the addition of Alexa Fluor 488-conjugated anti-rabbit IgG (green). One representative immunofluorescence image out of 3 independent replicates are shown; scale bars: 10 μm; **(B)** Representative transmission electron microscopy images of *M. aurum* infected RAW264.7 cells treated with 10 μM RNase3 or 10 μM RNase3-H15A. Autophagic vacuoles are indicated by white arrow; **(C)** Quantitative analysis of LC3 intensity normalized with cell volume from 3 replicates. Results are shown from 3 independent experiments (mean ± SD). *Statistically significant differences compared with the control sample are indicated (*p* value ≤ 0.05). **(D)** total number of autophagic vacuoles per cell for 30 cells randomly selected per each treated sample. Autophagic vacuoles were defined as double-membrane vacuolar structures containing recognizable cytoplasmic contents. *Statistically significant differences compared with the control sample are indicated (*p*-value ≤ 0.05).

In addition, LC3 transformation was monitored by immunofluorescence microscopy after treatment of *M. aurum* infected RAW macrophage cells with either RNase3, RNase3-H15A, or rapamycin, used as a positive control of autophagy induction, observing in all cases a significant increase of LC3 intensity (Figures 5A,C). Complementarily, we applied TEM to visualize the ultrastructure of mouse macrophages infected with *M. aurum* and treated with RNase3 and RNase3-H15A. Inspection of electron micrographs revealed a higher number of double-membrane vacuoles in both RNase3 and RNase3-H15A treated macrophages in comparison with the control group (Figures 5B,D). Thus, the enzymatic activity is not been involved in the RNases autophagy induction process.

### The Protein Autophagy Induction Correlates With Vesicles Agglutination Activity

Accumulation of double-membrane vacuoles, associated to the autophagosome formation, constitutes a characteristic trait of the autophagy pathway (36). Together with the initial autophagic vacuoles we could also identify abundant single-membrane degradative vacuoles, that could be ascribed to autolysosomes and are characteristic of the later steps of the autophagy process (Figure 5B). Moreover, macrophage incubation with RNase3 presented a dose dependent increase in the total volume of acidic vesicular compartments, as quantified by registering the AO acidotropic dye signal, a trait associated to autolysosome formation (Figure S6). In contrast, no significant change in AO signal was recorded for the none-autophagic RNase7, used here as a negative control. Rapamycin was used as a positive control and addition of BA, an inhibitor of the vacuolar H^+^ -ATPase, resulted in a significant reduction of the AO signal.

To note, the two RNases that activate autophagy, RNase3 and RNase6, can agglutinate liposome vesicles, in contrast to RNase7 (Figure S7). Likewise, RNases 3 and 6, but not RNase7, have mycobacteria cell agglutination activity (Table 1). The results suggest that the protein agglutination activity might be involved in the autophagy induction process.

### Induction of Macrophage Autophagy Contributes to RNase Antimycobacterial Activity

**Figure 6:**
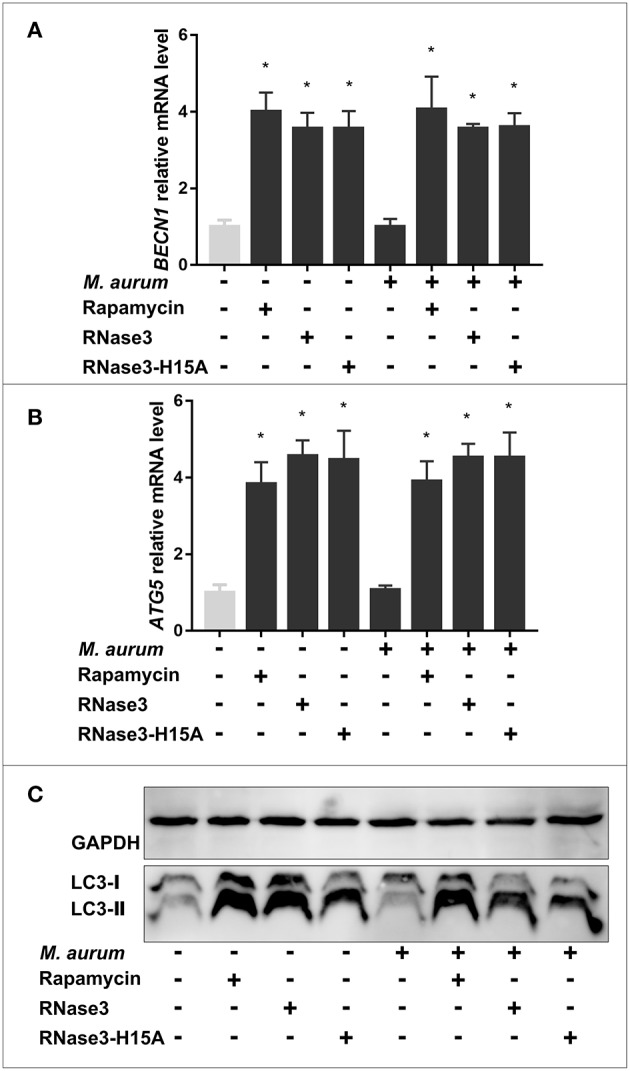
RNase3 and RNase3-H15A induce autophagy in control and *M. aurum* infected macrophages. Mouse macrophages were uninfected or infected with *M. aurum* and treated with 10 μM of protein (RNase3, RNase3-H15A) or 100 nM Rapamycin for 24 h. Real-time qPCR measured the relative expression of **(A)** *BECN1* and **(B)** *ATG5* genes normalized by the housekeeping gene β*-actin*. Results are shown from 3 independent experiments (mean ± SD); **(C)** Immunoblot of LC3-processing and the housekeeping GAPDH control. Results are shown from 3 independent experiments (mean ± SD). *Statistically significant differences compared with the control sample are indicated (*p-*value < 0.05).

Next, the induction of the autophagy process was confirmed for wild-type and mutant proteins in both infected a non-infected macrophages. The levels of the autophagy markers in RNase3 treated samples were also compared to rapamycin, observing in all cases comparable levels of *BECN1* and *ATG5* expression and LC3II/LC3I ratio (Figure 6). Induction of autophagy by RNase3 was reproduced on both control and infected macrophages (Figure 6). Results also corroborated that there were no significant differences between the effect induced by wild-type RNase3 and the H15A mutant variant on both infected and non-infected macrophage populations.

**Figure 7:**
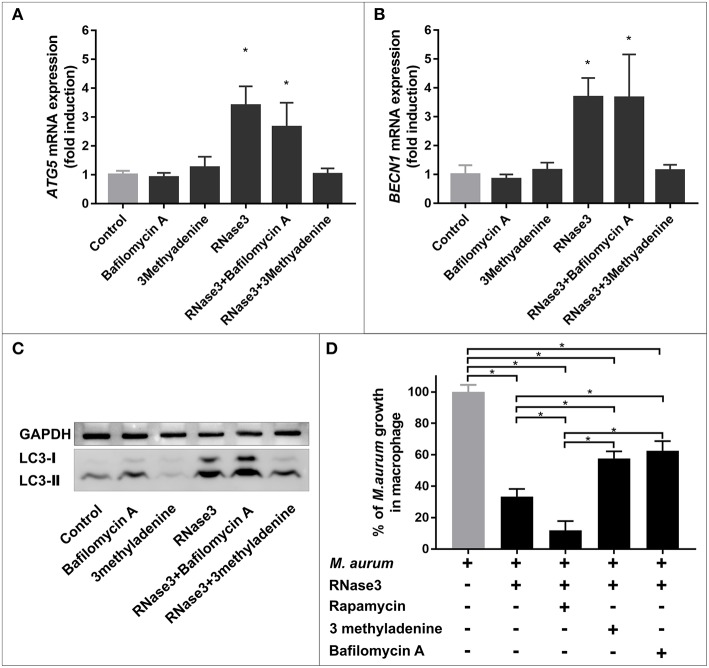
Autophagy induction by RNase3 contributes to its antimycobacterial activity against *M. aurum*. Mouse macrophages were infected with *M. aurum* and treated with 5 μM of RNase3 and/or 100 nM of rapamycin, and/or 100 nM of bafilomycin A1, and/or 5 mM of 3 methyladenine for 24 h. Real-time qPCR measured the relative expression of *BECN1* **(A)** and *ATG5* **(B)** genes normalized by the housekeeping gene β*-actin*. Results are shown from 3 independent experiments (mean ± SD); **(C)** A representative immunoblot of LC3 and housekeeping GAPDH control; **(D)** Intracellular mycobacterial viability was determined based on the number of CFUs. Relative percentages to control samples are indicated. Results are shown from 3 independent experiments (mean ± SD). *Significant differences between pairs is indicated (*p* value < 0.05).

**Figure 8:**
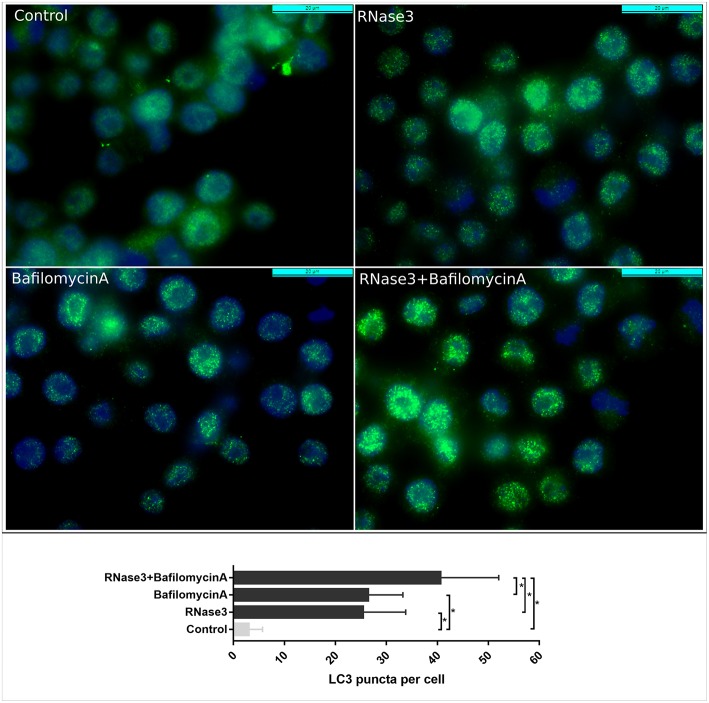
Comparative analysis by immunofluorescence microscopy of LC3 accumulation following RNase3 treatment in the absence and presence of Bafilomycin A. Mouse RAW264.7 cells were incubated with 10 μM RNase3 and/or 100 nM of Bafilomycin A1. Primary anti-LC3 antibody and Alexa-488 labeled secondary antibody were used to detect LC3 (green). Nuclei were labeled with DAPI (blue). Following immunostaining, 100 cells from each treatment were analyzed and the number of LC3 puncta per cell was calculated by *Image J*. *Statistically significant differences are indicated, *p* < 0.05.

Following, we evaluated whether the RNase induction of autophagy mediates the protein macrophage intracellular killing of mycobacteria. First, the levels of autophagy markers (*BECN1, ATG5*, and LC3-II) were quantified following RNase3 treatment in the absence and presence of autophagy inhibitors (3methyladenine and bafilomycin A1). 3MA is an early inhibitor of the autophagy pathway and works by blocking the autophagosome formation, whether BA arrest the autophagic process in a later step by impeding the autophagosome maturation and subsequent fusion with the lysosome compartment. Accordingly, while the presence of 3MA blocked the RNase3 induction of autophagy, no reduction in the expression levels of the autophagy markers were observed in the samples treated with BA (Figures 7A–C). In fact, quantification of LC3 accumulation by immunofluorescence microscopy revealed a significant increase of LC3 puncta per cell when macrophages were incubated with RNase3 in the presence of BA (Figure 8).

More interestingly, both autophagy inhibitors reduced significantly the protein ability to kill mycobacteria within macrophages (Figure 7D, Figure S8). The fact that 3MA inhibits RNase3 induction of autophagy, but not BA treatment, suggests that the protein triggers the onset of the autophagy process. Moreover, BA reduced the protein ability to kill the intracellular mycobacteria, indicating that the RNase anti-infective activity is partly dependent on the maturation of the autophagosome. Likewise, rapamycin co-treatment with RNase3 enhanced the protein antimicrobial effectivity (Figure 7D). An equivalent pattern was reproduced by the RNase3-H15A mutant (Figure S8). Therefore, the results confirm that the induction of autophagy participates in the RNase3 antimycobacterial activity within infected macrophages.

## Discussion

Previous studies have demonstrated that macrophages serve as a major protecting niche for dormant mycobacteria that can trigger a later reactivation from the disease latency. This scenario is one of the main hindrances in developing effective eradication of tuberculosis. Antimicrobial peptides participate in the host innate immunity against macrophage dwelling TB bacilli (12). Currently, the fight against emergent MDR-TB-forms is at the top priority of global health action programs (1). Within this context, the search for novel anti-TB agents is a priority. In this work, we have taken profit of our developed screening platform adapted for testing the drug efficacy within infected macrophage cells. The SPOTi high-throughput screening (HTS) assay system uses a non-pathogenic fast-growing mycobacterial species (*M. aurum*) able to survive within RAW 264.7 mouse macrophages, as a surrogate model for host TB infection. *M. aurum* is an appropriate substitute for *M. tuberculosis* among other non-pathogen mycobacteria candidates sharing a similar cell-wall structure, gene organization, and drug resistance pattern (8, 26, 27). The SPOTi assay was applied to screen the seven main canonical human members of the RNase A superfamily. The human canonical RNases, although sharing a high sequence identity and a conserved structural fold (Figure S9), differ considerably in their biological properties. Overall, RNases are secreted by different types of innate immune cells and participate in a variety of endogenous processes involved in the host defense (18, 28, 37, 38). One of the main ascribed tasks for human secretory RNases is the safeguard of the body fluid sterility. A diversity of anti-infective activities have been reported for some family members (18, 39–42). Among them, the protein mechanism of action on bacterial cells is probably one of the best studied processes (37, 38, 43). In particular, previous works identified the involvement of RNases 3 and 7 in mycobacterial infection. Expression of RNase3 together with α-defensin in *M. bovis* BCG infection was first reported by Capron and coworkers (14). Interestingly, the researchers observed the specific recruitment and induction of eosinophils by lipomannan, a unique mycobacterial cell-wall component. Very recently, Rivas-Santiago and coworkers reported the overexpression of RNase7 in airway epithelial cells infected by *M. tuberculosis* (24). In our laboratory we have confirmed the antimycobacterial activity of recombinant RNase3 and RNase7 on *M. vaccae in vitro* and characterized the protein mechanism of action at the bacterial cell-wall (25).

In this work, we have screened the antimycobacterial activity of the seven main canonical RNases. Taking advantage of the SPOTi-HTS, the protein activities were simultaneously assayed *in vitro* and *ex vivo* (8, 26, 27). Results indicated that out of the seven tested human RNases only three were active against both extracellular and intracellular macrophages-dwelling mycobacteria (Figures 1, 2). A high antimycobacterial activity was observed for RNases 3, 6 and 7, while RNases 1, 2, 4 and 5 were found inactive even at the highest concentration tested. The three active RNases (RNases 3, 6, and 7) correspond to the family members with previously reported antimicrobial activity against Gram-negative and Gram-positive species (37, 38, 41). The present data corroborate that RNase3 is the most active among the family members.

Following, the three active RNases (RNases 3, 6, and 7) were selected for further analysis. First, the minimum bactericidal activity was determined *in vitro* against three distinct *Mycobacterium* species: *M. smegmatis* mc^2^155, *M. aurum* and *M. bovis* BCG (Table 1). In addition, the protein cell agglutination activity was tested against the three assayed mycobacteria species (*M. smegmatis* mc^2^155, *M. aurum*, and *M. bovis* BCG). Interestingly, RNases 3 and 6 were able to induce the aggregation of all the tested mycobacteria, but not RNase7 (Table 1). The results are in accordance with our previously published data using *M. vaccae* cultures, where RNase3, but not RNase7, showed a significant mycobacterial cell agglutination activity (25). Previous works from our laboratory on Gram-negative species also revealed the agglutination ability of RNases 3 and 6 in contrast with RNase7 (21, 38). Likewise, RNases 3 and 6 can agglutinate phospholipid vesicles but not RNase7 (Figure S7) (20, 38). Positive agglutination was correlated with the presence of an aggregation-prone hydrophobic patch within the primary sequences of RNases 3 and 6 (37, 44–46). Comparative scanning of the three RNases profiles using the AGGRESCAN 3D software (47) also highlighted the absence of an aggregation patch at the RNase7 structure surface (Figure S9). On the other hand, no significant differences were observed between the wild-type and H15A mutants of the three RNases showing antimycobacterial activity (Table 1). The present results on mycobacteria confirm previous studies on Gram-negative and Gram-positive bacterial species that indicated that the RNases catalytic activity did not participate in their antimicrobial action (37, 38, 48–51).

To evaluate the potential translational significance of our results in an *in vivo* scenario, we analyzed the expression levels of the RNases in human macrophage THP1 cells following *M. aurum* infection. To this end, we synthesized primers for the four human family members with described anti-pathogen activities and previously reported to be expressed in granulocytes (18). Our data highlighted that the human monocyte-derived THP1 macrophages primarily expresses RNase2 and secondarily RNase3 and RNase6, while a very scarce expression of RNase7 is detected (Figure S5). This expression pattern corroborates previous studies that highlighted a predominant expression of RNase2 in human monocytes. On the other hand, RNase7 was reported to be abundantly expressed in epithelial cells (52) but poorly in macrophages (23). In addition, Becknell and co-workers reported a regulation of RNase6 expression in macrophages in response to uropathogenic *E. coli* infection (23). The present data indicates that *M. aurum* infection is only altering the expression profile of cultured macrophages for *RNase3* and *RNase6* genes. However, further work would be needed to characterize the RNases expression pattern in primary macrophages. The results are in accordance with the previously observed anti-pathogen properties of the three detected RNases. RNase2 has a high antiviral activity but no antibacterial activity *in vitro*, and is regulated during viral infection (40). On their turn, RNases 3 and 6 have a high bactericidal activity and their expression is regulated by bacterial infections (18, 23, 42). The present time-course expression profiles in *M. aurum* infected THP1 macrophages indicates a downregulation of RNase3 and RNase6 after a short infection period followed by a significant upregulation at longer incubation times. Modulation of antimicrobial RNases following *M. aurum* infection are in agreement with previously reported results using *M. tuberculosis* (18, 24). In the literature there are other examples of AMPs downregulated upon mycobacteria infection, a process induced by the Mtb bacilli as a protection mechanism against the host innate armory (12, 30). On its turn, upon infection, the host immune cells activate the expression of antimicrobial proteins and peptides (12, 53). Many stimuli have been reported to regulate the expression of human RNases, such as bacterial, viral or parasite infection together with inflammation, sepsis and tissue damage (18, 42, 54). Intriguingly, the comparative expression pattern of the distinct RNaseA members reveals a clear specialization in regard to innate cell and infection types (18, 55). In this context, RNase7 is mainly secreted by epithelial tissues and conforms a protection barrier against bacterial intruders, whereas RNases 3 and 6 are abundant in blood cell types. In particular RNase 3 is highly expressed in eosinophils while RNase6 predominates in neutrophils and monocytes (18). In addition, free secretory granules from eosinophils and neutrophils can be engulfed by macrophage cells and release their content within (18, 56, 57).

Our present results using murine RAW macrophages infected with *M. aurum* highlighted that the three antimicrobial RNases can effectively eradicate the macrophage intracellular resident mycobacteria at a 5 to 10 μM range (Table 1). The fact that the RNases could more easily eradicate the mycobacteria resident within macrophages than free in extracellular cultures suggested us that the proteins might combine a direct bacterial cell killing action with some other biological properties.

Immunomodulatory activities mediate the antimycobacterial action of many AMPs (12, 58–60). One of the main strategies undertaken by AMPs to control the proliferation of macrophage intracellular resident bacteria relies on the triggering of the autophagy pathway (12, 30, 34, 61–63). Selective autophagy targeting invading pathogens contributes to maintain the host tissue homeostasis (64). On its turn, the Mtb bacilli ensure their intracellular survival within macrophages by arresting the autophagosome maturation (65, 66). Therefore, autophagy inducers are among the favorite drug candidates against resistant TB strains (12, 34, 58, 59, 62, 67). Interestingly, out of the seven screened RNases, RNase3, and RNase6 significantly increased *BECN1* and *ATG5* gene expression and LC3 processing was observed (Figure 3). Subsequent analysis using RNase3 as a reference indicated that the autophagy induction process is unrelated to the protein catalytic activity (Figures 4, 5). Moreover, the autophagy induction was reproduced in both infected and non-infected macrophages (Figure 6).

The contribution of autophagy induction on the RNase3 antimycobacterial activity was corroborated by evaluating the protein effect on *M. aurum* growth upon blockage of the autophagy pathway. The blockage of the autophagosome formation or maturation was induced by either addition of 3MA, an inhibitor of the PI3K class III kinase (68), or BA, an ATPase inhibitor that prevents the lysosome fusion with the autophagic vesicles (69). When the infected macrophages were treated with RNase3 in the presence of either 3MA or BA we observed a reduction in the protein inhibitory activity of *M. aurum* growth (Figure 7 and Figure S8). On the other hand, rapamycin enhanced the antimycobacterial activity of RNase3. The same pattern was reproduced when assaying the RNase3-H15A mutant action in the presence of the autophagy inhibitor or activator (Figure S8).

The fact that BA does not inhibit the RNase induction of autophagy but reduces its antimycobacterial activity suggests that the protein action is dependent on the late autophagosome maturation process. Moreover, upon cell incubation with RNase3 we observed an increase in the acidic vesicle content (Figure S6), a process that can be associated to autolysosome formation. To note, other AMPs, such as azurodicin and calgranulin, activate autophagy by promoting the autophagosome fusion with lysosomes (12, 53, 70, 71). Overall, our results confirm that autophagy contributes to the RNase antimycobacterial mechanism of action. However, the fact that addition of the autophagy inhibitors does not fully abolishes the antimicrobial activity of the protein indicates that other biological properties are also participating in its antimycobacterial activity. In addition, considering that RNases 3 and 6 induce autophagy, but not RNase7, and that no significant differences are observed between the wild type and the active site mutant proteins, we can suspect that the protein aggregation-prone ability might be involved in the autophagy pathway. Indeed, our previous results correlated the protein self-aggregation capacity with the proteins lipid vesicle agglutination and membrane disruption ability (20). We can speculate that the formation of protein aggregates could promote the autophagosome formation and later fusion with the lysosomal compartment. Complementarily, other protein properties, such as high cationicity, were considered to underlie the autophagy process. The predicted isoelectric point (pI) for all tested seven RNases, ranging from 9 to 10.7 (Table S2), emphasizes their prominent cationicity, a characteristic trait of AMPs (72–74). However, although RNase3 is the most cationic of all the proteins, we cannot find a direct correlation between the pI and antimicrobial or autophagy induction abilities among the seven tested human RNases. Likewise, the protein hydrophobicity was analyzed, obtaining very similar calculated GRAVY values (Table S2). Further work is ongoing to unravel the structural determinants underlying the RNase antimycobacterial mechanism of action.

Interestingly, two RNaseA family homologs (bovine seminal RNase and onconase) were recently reported to exert their anti-tumoral activity by the selective induction of the autophagic cancer cell death (33, 75). Bovine seminal RNase (BS-RNase) triggers an autophagic cell death process in pancreatic cancer cells mediated by *BECN1* induction; and onconase, a RNaseA superfamily member proposed for chemotherapy, inhibits the proliferation of cancer cells through the ROS/Akt/mTOR pathway (33, 75). Surprisingly, the bovine pancreatic RNaseA, displaying a high catalytic activity and sharing about a 82% sequence identity with BS-RNase, could not reproduce the induction of autophagy. The authors associated the activation of the autophagic pathway to the protein cationicity and oligomer formation, discarding any correlation between the RNase catalytic activity and the autophagic process (75). Two main factors, protein net charge, and stability of the oligomers were considered to explain the difference between RNaseA and BS-RNase in inducing autophagy. Last but not least, there is another human RNase, the RNase L, unrelated to the RNaseA superfamily, which can also trigger the autophagy pathway. RNase L expression is induced during viral infection. The protein can initiate autophagy and suppress thereby the virus replication (31, 76). In all cases, we can outline a convergent mechanism where the RNases induction of autophagy is activated by a cell stress condition: oxidative injury, presence of protein aggregation, or infection (18, 77).

In summary, the present results have revealed the potentiality of human secretory RNases to work as antimicrobial agents in the fight against tuberculosis, extendable to other intracellular infectious bacterial diseases that are difficult to eradicate (78). AMPs that display a multifaceted mechanism of action (12, 16, 79–85), like human antimicrobial RNases, deserve a special attention for their pharmacological potential. Our ongoing inter-disciplinary investigation warrants the understanding of RNases' antimicrobial mechanism of action and can pave the design of novel anti-infective therapies.

## Conclusions

Screening of the seven main human canonical members of the RNaseA superfamily identified three RNases (RNase3, RNase6, and RNase7) that achieved the eradication of *M. aurum* infection within macrophages at a low micromolar range. The protein antimicrobial activity was found not dependent on the RNase enzymatic activity. Out of the three antimycobacterial RNases, two of them (RNase3 and RNase6) demonstrated their ability to induce macrophage autophagy and thereby inhibit *M. aurum* intracellular growth. The expression of both RNases in human THP1 derived macrophage cells is regulated by the mycobacterial infection, suggesting an *in vivo* physiological role.

## Data Availability

The raw data supporting the conclusions of this manuscript will be made available by the authors, without undue reservation, to any qualified researcher.

## Author Contributions

EB and SB contributed in funding acquisition. DP, EB, and SB conceived and designed the experiments. LL, GP-E, and JA-T performed the experiments. EB, GP-E, DP, and SB supervised the experimental work. LL, JA-T, EB, and SB contributed to the original draft preparation. LL, DP, EB, and SB participated in the manuscript revision and final edition.

### Conflict of Interest Statement

The authors declare that the research was conducted in the absence of any commercial or financial relationships that could be construed as a potential conflict of interest.

## Supplementary Material

Figure S1 A representative SPOTi image comparing human RNases activity on *M. aurum* cultures. *M. aurum* was incubated with serially diluted recombinant human RNases (ranging from 2 to 32 μM) for 4 h in phosphate-buffered saline (PBS). Next, an aliquot was spotted onto wells of a 24-well plate containing MB7H10/OADC/agar and incubated at 37°C for 4–5 days to determine survival.

Figure S2 Confocal microscopy analysis of RAW 264.7 cell culture (2.5 × 10^5^ cells/mL) incubated with 2 μM of RNase3 labeled with Alexa Fluor 488. Cells were stained with Hoechst and Deep Red following the assay incubation conditions detailed in the experimental procedures section. After addition of Alexa Fluor 488 labeled protein (green), the evolution of the fluorescence signals was analyzed by confocal microscopy. A total of 20 cells were analyzed by regions of interest (ROIs) using Leica TCS software. The images were taken using a Leica TCS SP5 AOBS microscope.

Figure S3 3D reconstruction of RAW 264.7 macrophages post-treatment with RNase3. Confocal microscopy analysis of RAW 264.7 cell culture incubated with 2 μM of RNase3 labeled with Alexa Fluor 488 (green). Cells were stained with Hoechst and Deep Red following the assay incubation conditions detailed in the experimental procedures section. After 45 min of protein addition, the fluorescence signals of Hoechst and AlexaFluor **(A)** and Hoechst, AlexaFluor and Deep Red **(B)** were analyzed by confocal microscopy. The images were taken using a Leica TCS SP5 AOBS microscope.

Figure S4 Confocal microscopy analysis of RAW 264.7 cell culture incubated with human RNases labeled with Alexa Fluor 488. Cells were stained with Hoechst and Deep Red following the assay incubation conditions detailed in the experimental procedures section. After labeled protein addition (green), the evolution of the fluorescence signals was analyzed by confocal microscopy for 30 min. A total of 20 cells were analyzed by regions of interest (ROIs) using Leica TCS software. The images were taken using a Leica TCS SP5 AOBS microscope. Each panel indicates the RNase assayed.

Figure S5 Expression pattern of human RNases in THP-1 derived macrophage cells infected with *M. aurum*. Human THP-1 macrophage derived cells were infected with *M. aurum* for 4, 24, 48, and 72 h. The transcriptional expression of human *RNase2, RNase3, RNase6*, and *RNase7* were detected by real-time qPCR and all samples were normalized with the GAPDH housekeeping gene. **(A)** relative expression of *RNase2, RNase3, RNase6*, and *RNase7* at 0 h without infection; **(B–D)** The monitoring of RNases' expression upon infection was presented as the fold change of relative expression in infected group compared with the corresponding control group at each time point. Results are shown from 3 independent experiments (mean ± SD), ^*^indicates a significant difference compared with control (*p* value < 0.05).

Figure S6 Quantification of acidic vesicular compartment measured by AO staining. The total amount of acidic vesicles was assessed as an estimate of autolysosome formation. Mouse RAW 264.7 macrophage cells were treated with RNases 3 and 7 for 24 h. Values are presented as means ± SD of 8 replicates. ^*^indicates significant difference compared with control group (*p* ≤ 0.05).

Figure S7 Comparison of aggregation of DOPG/DOPC liposomes by RNases. The incubation time of protein with liposomes is 30 min for all assay. The proteins were 2-fold serially diluted (from 1.6 to 0.05 μM).

Figure S8 RNase3 and RNase3-H15A antimycobacterial activity in the presence of autophagy regulators. RAW 264.7 macrophages were infected with *M. aurum* and treated with different concentration of the proteins, and/or 100 nM of rapamycin (Rapa), and/or 100 nM of bafilomycin A (BA), and/or 5 mM of 3 methyladenine (3MA) for 24 h in RPMI-1640 complete medium. Macrophages were washed twice with RPMI-1640 and lysed, and then an aliquot was spotted onto wells of a 24-well plate containing MB7H10/OADC/agar and incubated at 37°C for 4–5 days to determine intracellular survival.

Figure S9 Sequence alignment and prediction of aggregation propensity of eight human RNases. **(A)** Alignment of the eight human canonical RNases using the ESPript3 software (espript.ibcp.fr/), the N-terminal domain is highlighted in blue. Sequence allocation was performed following the homology between RNases. **(B)** 3D structure of RNases by *Aggrescan3D* (*A3D*). *A3D* exploits an experimentally derived intrinsic aggregation propensity scale for natural amino acids. This structure-based approach identifies aggregation patches (in red) at the protein surface. **(C)** Aggregation profile propensity of RNase3, RNase6, and RNase7 based on *A3D* score for protein residues.

Table S1 Primer sequences for real-time qPCR. Primers of human *GAPDH, RNase2, RNase3, RNase6*, and *RNase7*, and human and mouse β*-actin, Beclin-1, and ATG5*.

Table S2 Comparison of physicochemical parameters of human secretory RNases. pI and Grand average of hydropathy (GRAVY) were predicted using the ProtParam tool (https://web.expasy.org/translate/).

## Abbreviations

DOPC: 1,2-dioleoyl-sn-glycero-3-phosphocholine
DOPG: 1,2-dioleoyl-sn-glycero-3-phosphoglycerol)
3MA: 3 Methyadenine
MTT: 3-(4,5-Dimethylthiazol-2-yl)-2,5-diphenyltetrazolium bromide
AO: Acridine orange
AMPs: Antimicrobial protein and peptides
BA: Bafilomycin A
CFU: Colony forming unit
XDR: Extensively drug-resistant
FBS: Fetal bovine serum
HTS: High-throughput screening
LUVs: Large unilamellar vesicles
MB: Middlebrook
MAC: Minimum agglutination activity
MBC: minimum bactericidal concentration
MIC: minimum inhibitory concentration
MOI: Multiplicity of infection
MDR: Multi-drug resistant
Mtb: *Mycobacterium tuberculosis*
NCTC: National Collection of Type Cultures
PMA: Phorbol myristate acetate
SPOTi: Spot-culture growth inhibition assay
TEM: Transmission electron microscopy
TB: Tuberculosis
ZN: Ziehl-Neelsen.
