# Supplementary figures and images for: Human Antimicrobial RNases Inhibit Intracellular Bacterial Growth and Induce Autophagy in Mycobacteria-Infected Macrophages
Source: Front Immunol. 2019 Jul 2;10:1500. doi: 10.3389/fimmu.2019.01500 (PMC6614385; doi:10.3389/fimmu.2019.01500)

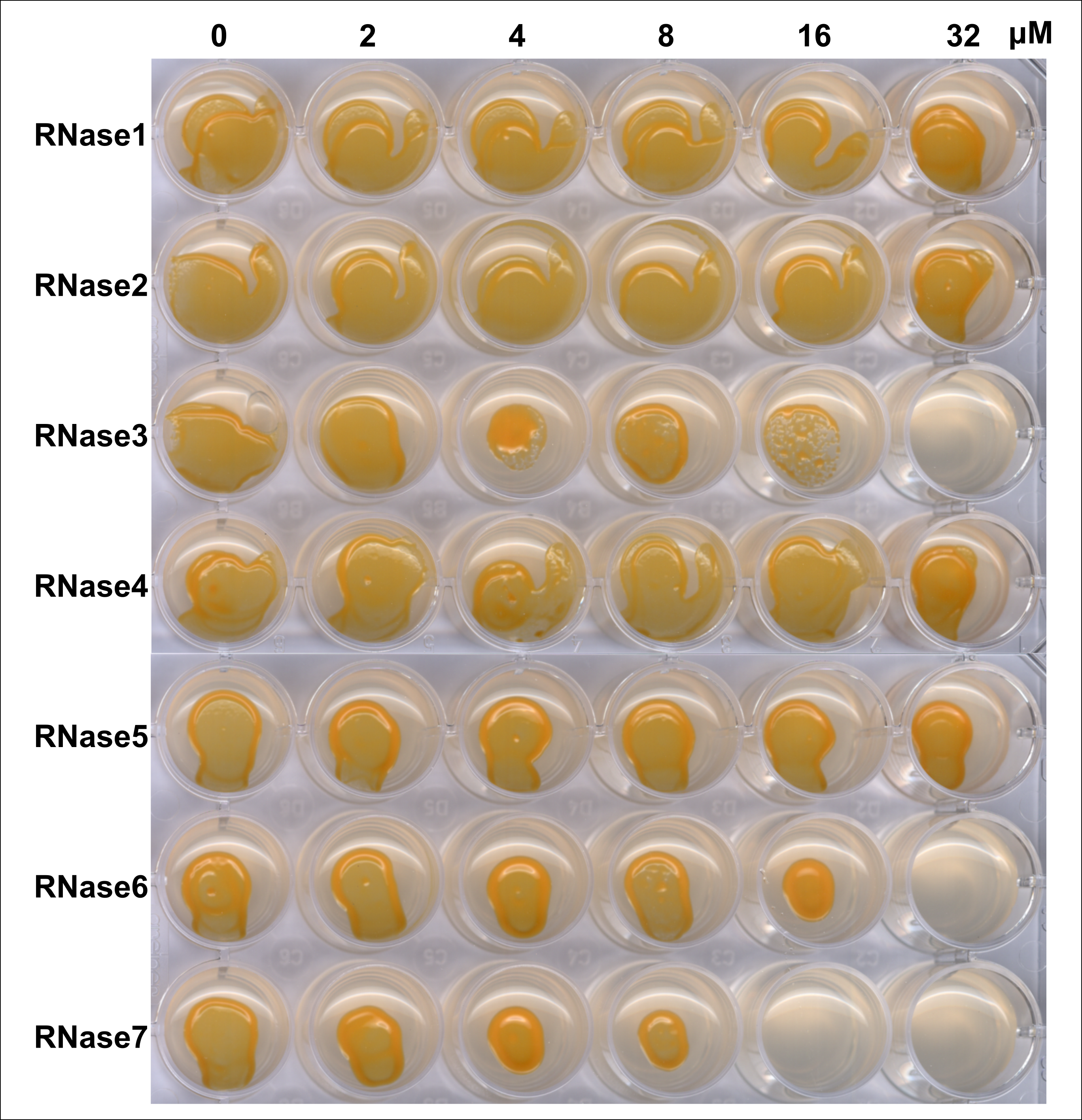

Supplement: Figure S1 — A representative SPOTi image comparing human RNases activity on M. aurum cultures. M. aurum was incubated with serially diluted recombinant human RNases (ranging from 2 to 32 μM) for 4 h in phosphate-buffered saline (PBS). Next, an aliquot was spotted onto wells of a 24-well plate containing MB7H10/OADC/agar and incubated at 37°C for 4–5 days to determine survival. [file Image_1.TIF]

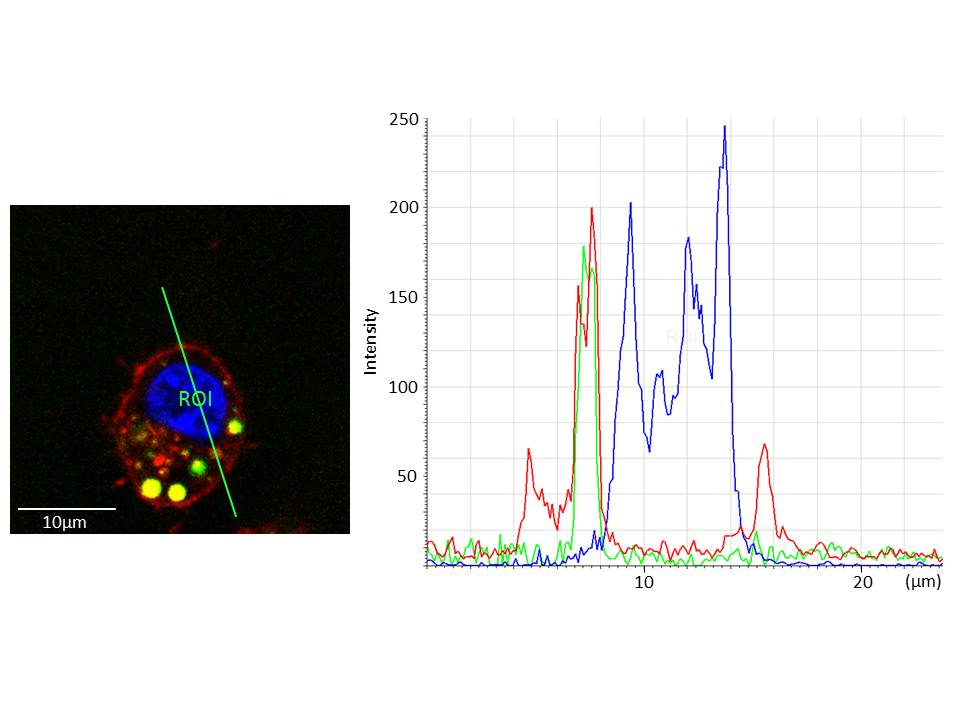

Supplement: Figure S2 — Confocal microscopy analysis of RAW 264.7 cell culture (2.5 × 10^5^ cells/mL) incubated with 2 μM of RNase3 labeled with Alexa Fluor 488. Cells were stained with Hoechst and Deep Red following the assay incubation conditions detailed in the experimental procedures section. After addition of Alexa Fluor 488 labeled protein (green), the evolution of the fluorescence signals was analyzed by confocal microscopy. A total of 20 cells were analyzed by regions of interest (ROIs) using Leica TCS software. The images were taken using a Leica TCS SP5 AOBS microscope. [file Image_2.TIF]

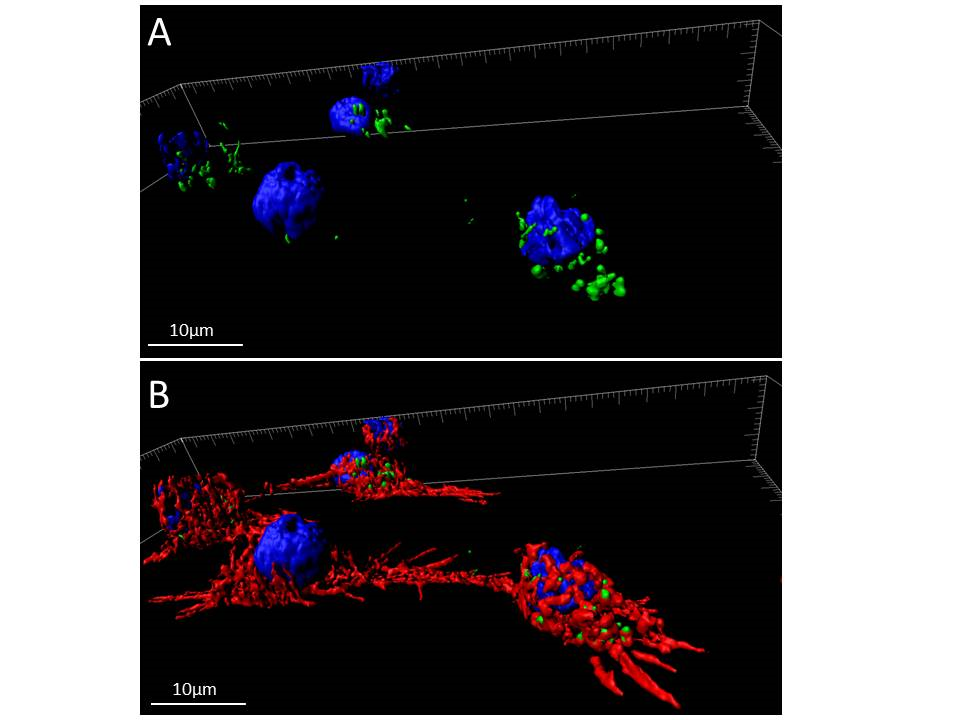

Supplement: Figure S3 — 3D reconstruction of RAW 264.7 macrophages post-treatment with RNase3. Confocal microscopy analysis of RAW 264.7 cell culture incubated with 2 μM of RNase3 labeled with Alexa Fluor 488 (green). Cells were stained with Hoechst and Deep Red following the assay incubation conditions detailed in the experimental procedures section. After 45 min of protein addition, the fluorescence signals of Hoechst and AlexaFluor (A) and Hoechst, AlexaFluor and Deep Red (B) were analyzed by confocal microscopy. The images were taken using a Leica TCS SP5 AOBS microscope. [file Image_3.TIF]

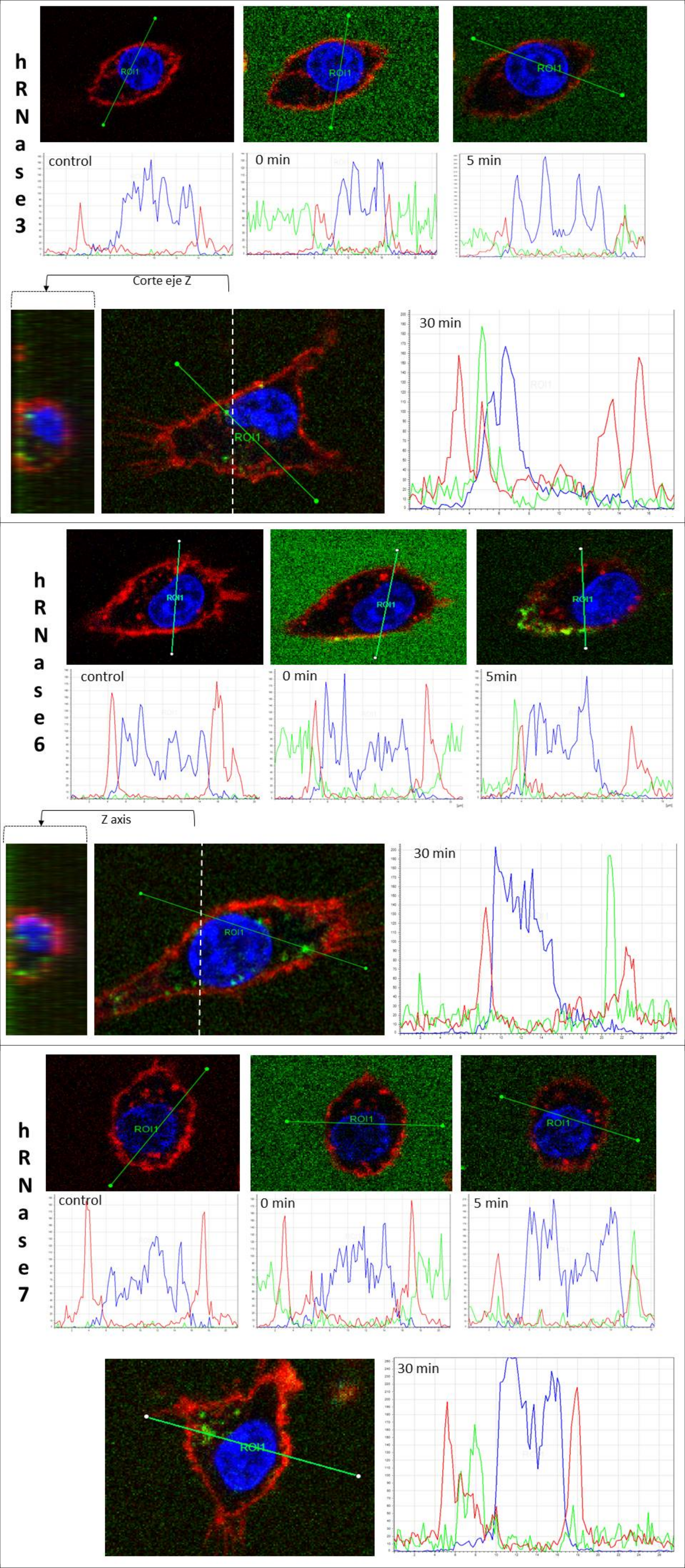

Supplement: Figure S4 — Confocal microscopy analysis of RAW 264.7 cell culture incubated with human RNases labeled with Alexa Fluor 488. Cells were stained with Hoechst and Deep Red following the assay incubation conditions detailed in the experimental procedures section. After labeled protein addition (green), the evolution of the fluorescence signals was analyzed by confocal microscopy for 30 min. A total of 20 cells were analyzed by regions of interest (ROIs) using Leica TCS software. The images were taken using a Leica TCS SP5 AOBS microscope. Each panel indicates the RNase assayed. [file Image_4.TIF]

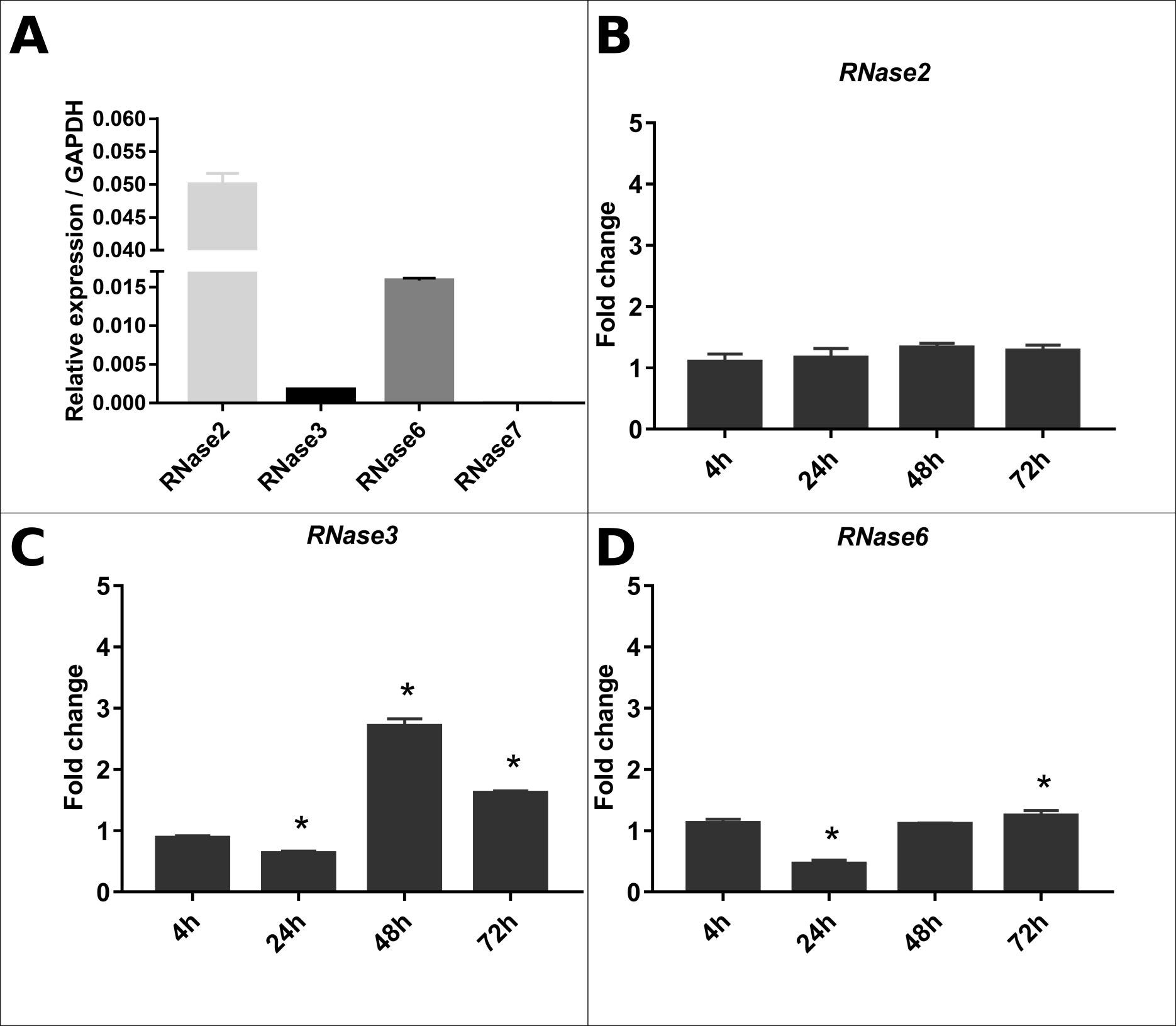

Supplement: Figure S5 — Expression pattern of human RNases in THP-1 derived macrophage cells infected with M. aurum. Human THP-1 macrophage derived cells were infected with M. aurum for 4, 24, 48, and 72 h. The transcriptional expression of human RNase2, RNase3, RNase6, and RNase7 were detected by real-time qPCR and all samples were normalized with the GAPDH housekeeping gene. (A) relative expression of RNase2, RNase3, RNase6, and RNase7 at 0 h without infection; (B–D) The monitoring of RNases' expression upon infection was presented as the fold change of relative expression in infected group compared with the corresponding control group at each time point. Results are shown from 3 independent experiments (mean ± SD), *indicates a significant difference compared with control (p value < 0.05). [file Image_5.TIF]

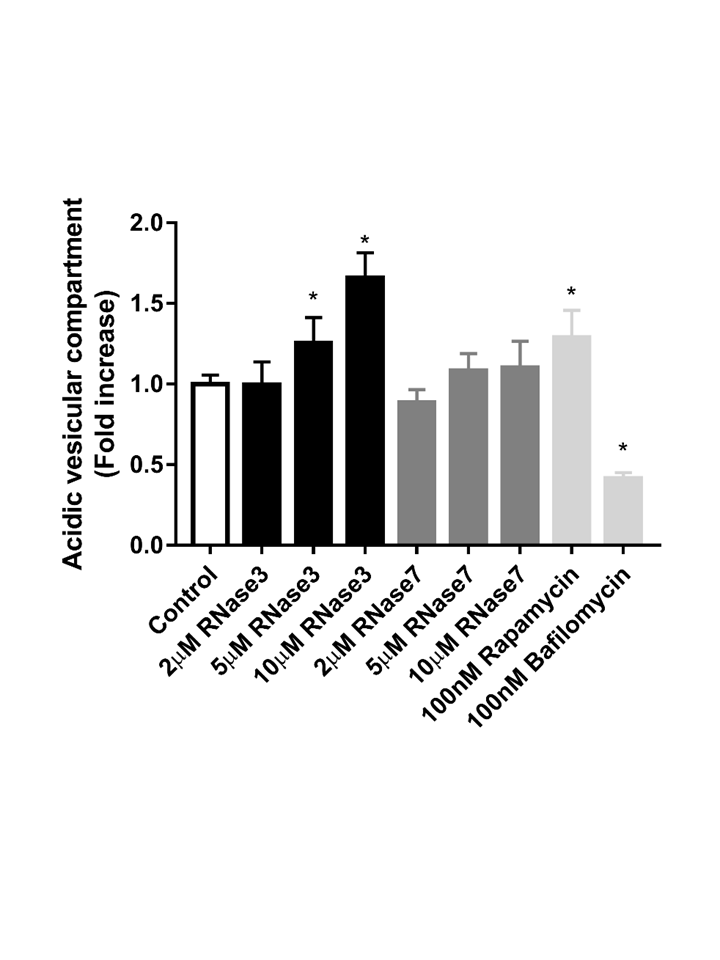

Supplement: Figure S6 — Quantification of acidic vesicular compartment measured by AO staining. The total amount of acidic vesicles was assessed as an estimate of autolysosome formation. Mouse RAW 264.7 macrophage cells were treated with RNases 3 and 7 for 24 h. Values are presented as means ± SD of 8 replicates. *indicates significant difference compared with control group (p ≤ 0.05). [file Image_6.TIF]

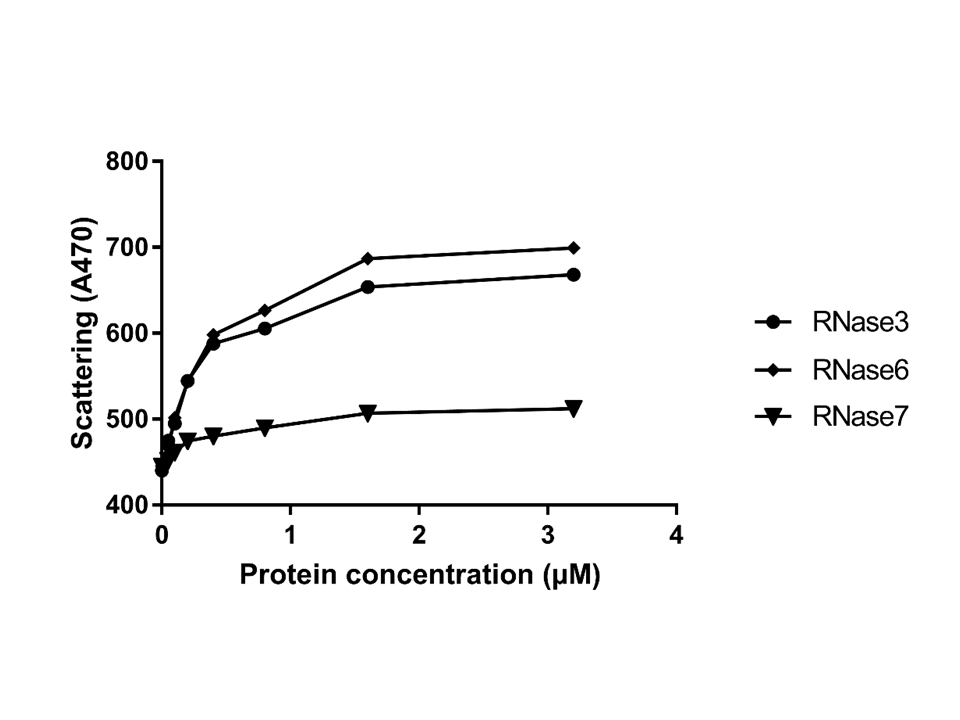

Supplement: Figure S7 — Comparison of aggregation of DOPG/DOPC liposomes by RNases. The incubation time of protein with liposomes is 30 min for all assay. The proteins were 2-fold serially diluted (from 1.6 to 0.05 μM). [file Image_7.TIF]

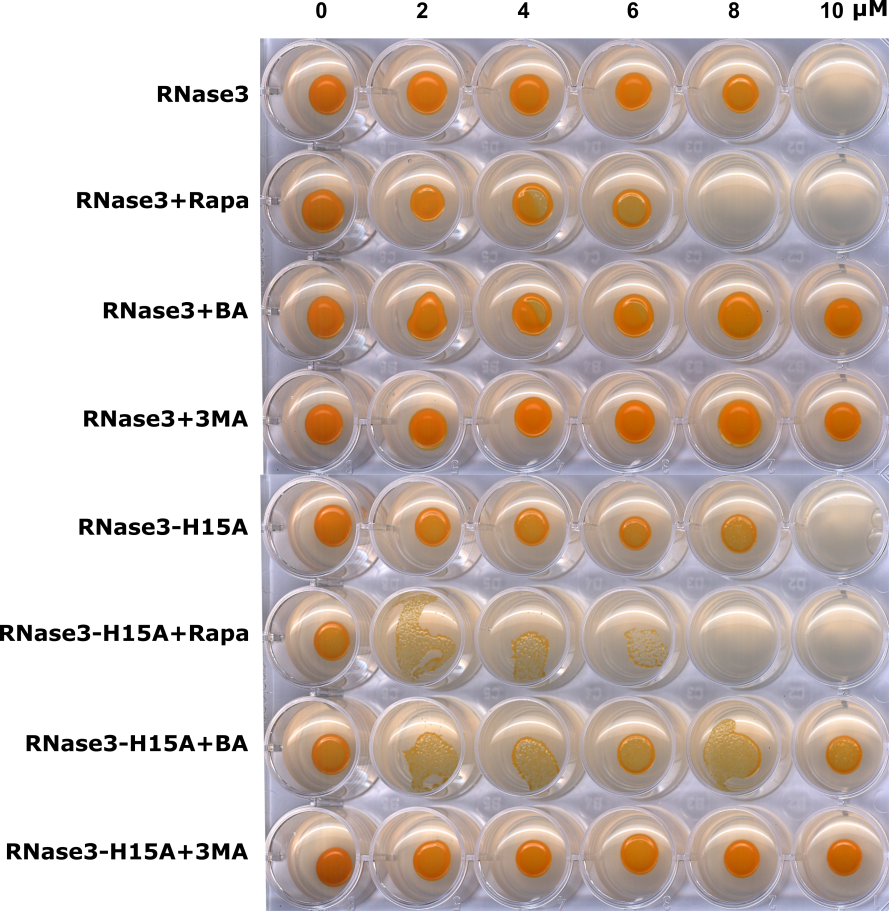

Supplement: Figure S8 — RNase3 and RNase3-H15A antimycobacterial activity in the presence of autophagy regulators. RAW 264.7 macrophages were infected with M. aurum and treated with different concentration of the proteins, and/or 100 nM of rapamycin (Rapa), and/or 100 nM of bafilomycin A (BA), and/or 5 mM of 3 methyladenine (3MA) for 24 h in RPMI-1640 complete medium. Macrophages were washed twice with RPMI-1640 and lysed, and then an aliquot was spotted onto wells of a 24-well plate containing MB7H10/OADC/agar and incubated at 37°C for 4–5 days to determine intracellular survival. [file Image_8.TIF]

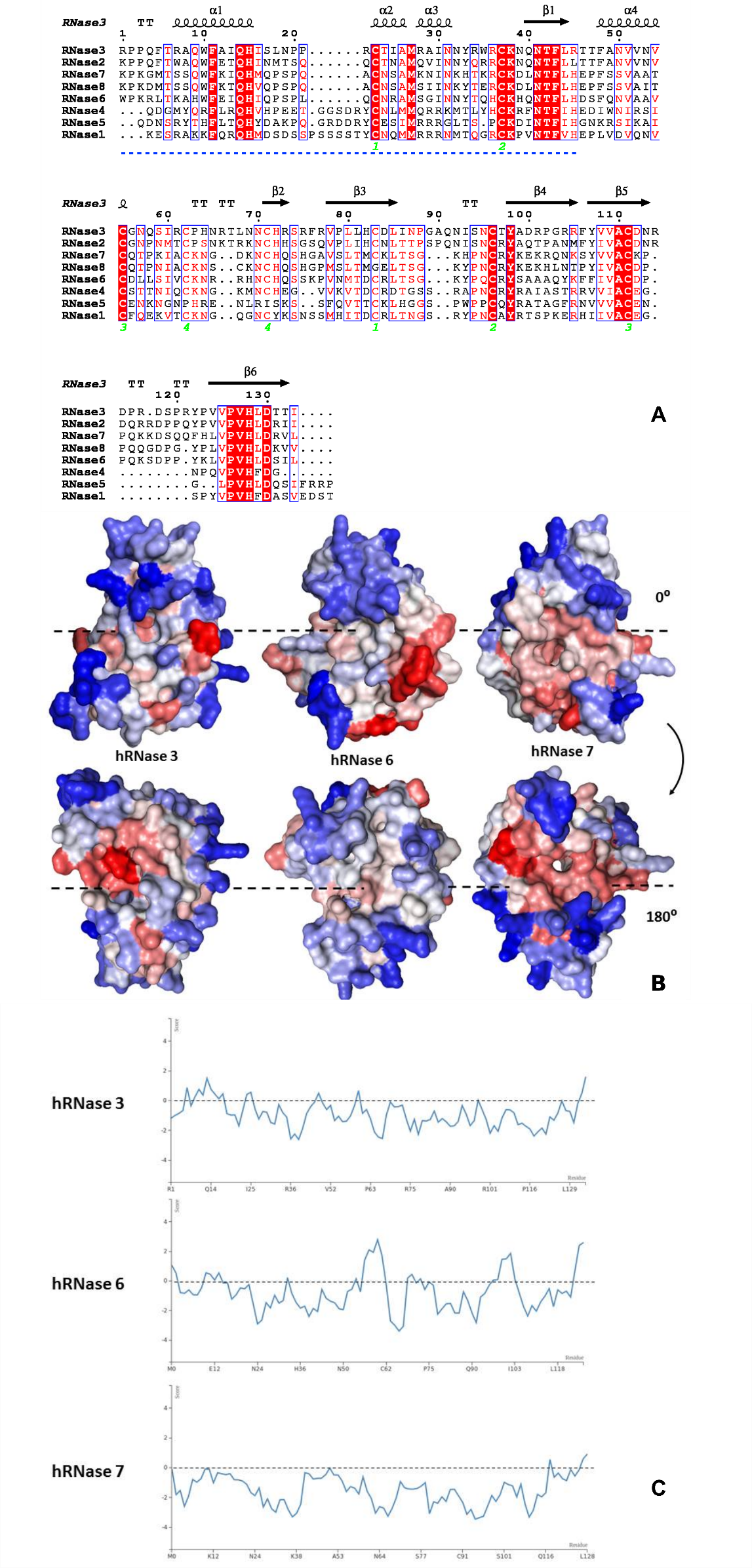

Supplement: Figure S9 — Sequence alignment and prediction of aggregation propensity of eight human RNases. (A) Alignment of the eight human canonical RNases using the ESPript3 software (espript.ibcp.fr/), the N-terminal domain is highlighted in blue. Sequence allocation was performed following the homology between RNases. (B) 3D structure of RNases by Aggrescan3D (A3D). A3D exploits an experimentally derived intrinsic aggregation propensity scale for natural amino acids. This structure-based approach identifies aggregation patches (in red) at the protein surface. (C) Aggregation profile propensity of RNase3, RNase6, and RNase7 based on A3D score for protein residues. [file Image_9.TIF]
