# Supplementary material for: Human Antimicrobial RNases Inhibit Intracellular Bacterial Growth and Induce Autophagy in Mycobacteria-Infected Macrophages
Source: Front Immunol. 2019 Jul 2;10:1500. doi: 10.3389/fimmu.2019.01500 (PMC6614385; doi:10.3389/fimmu.2019.01500)
Supplement: Table S1 — Primer sequences for real-time qPCR. Primers of human GAPDH, RNase2, RNase3, RNase6, and RNase7, and human and mouse β-actin, Beclin-1, and ATG5. [file Table_1.DOCX]

| **Specie** | **Gene** | **Sense** | **Antisense** | **Unique ID / Reference** |
| --- | --- | --- | --- | --- |
| Human | *GAPDH* |  |  | qHsaCED0038674 |
| Human | *RNase2* |  |  | qHsaCED0020010 |
| Human | *RNase3* |  |  | qHsaCED0001992 |
| Human | *RNase6* |  |  | qHsaCED0046630 |
| Human | *RNase7* |  |  | qHsaCID0020296 |
| Human | *β-actin* | ATCTGGCACCACACCTTCTACAATGAGCTGCG | ACACCAGACATAGTAGCAGAAATCAAG | (1) |
| Human | *ATG5* | TGGGATTGCAAAATGACAGA | TTTCCCCATCTTCAGGATCA | (1) |
| Human | *Beclin-1* | CCAGGATGGTGTCTCTCGCA | CTGCGTCTGGGCATAACGCA | (1) |
| Mouse | *β-actin* | GGCACCACACCTTCTACAATG | GGGGTGTTGAAGGTCTCAAAC | (2) |
| Mouse | *ATG5* | GACAAAGATGTGCTTCGAGATGTG | GTAGCTCAGATGCTCGCTCAG | (3) |
| Mouse | *Beclin-1* | GTGCTCCTGTGGAATGGAAT | GCTGCACACAGTCCAGAAAA | (4) |

**Table S1. Primer sequences for real-time qPCR.** Primers of human GAPDH, RNase2, RNase3, RNase6 and RNase7, and human and mouse *β-actin, Beclin-1 and ATG5*.

1. Yuk J-M, Shin D-M, Lee H-M, Yang C-S, Jin HS, Kim K-K, Lee Z-W, Lee S-H, Kim J-M, Jo E-K. Vitamin D3 Induces Autophagy in Human Monocytes/Macrophages via Cathelicidin. *Cell Host Microbe* (2009) **6**:231–43. doi:10.1016/j.chom.2009.08.004

2. Seldin MM, Lei X, Tan SY, Stanson KP, Wei Z, Wong GW. Skeletal Muscle-derived Myonectin Activates the Mammalian Target of Rapamycin (mTOR) Pathway to Suppress Autophagy in Liver. *J Biol Chem* (2013) **288**:36073–36082. doi:10.1074/jbc.M113.500736

3. Pua HH, Dzhagalov I, Chuck M, Mizushima N, He Y-W. A critical role for the autophagy gene Atg5 in T cell survival and proliferation. *J Exp Med* (2007) **204**:25–31. doi:10.1084/jem.20061303

4. Wang J-D, Cao Y-L, Li Q, Yang Y-P, Jin M, Chen D, Wang F, Wang G-H, Qin Z-H, Hu L-F, et al. A pivotal role of FOS-mediated BECN1/Beclin 1 upregulation in dopamine D2 and D3 receptor agonist-induced autophagy activation. doi:10.1080/15548627.2015.1100930
