# Supplementary material for: Human Antimicrobial RNases Inhibit Intracellular Bacterial Growth and Induce Autophagy in Mycobacteria-Infected Macrophages
Source: Front Immunol. 2019 Jul 2;10:1500. doi: 10.3389/fimmu.2019.01500 (PMC6614385; doi:10.3389/fimmu.2019.01500)
Supplement: Table S2 — Comparison of physicochemical parameters of human secretory RNases. pI and Grand average of hydropathy (GRAVY) were predicted using the ProtParam tool (https://web.expasy.org/translate/). [file Table_2.DOCX]

|  | RNase1 | | RNase2 | RNase3 | RNase4 | RNase5 | RNase6 | RNase7 |
| --- | --- | --- | --- | --- | --- | --- | --- | --- |
| Theoretical pI | | 8.98 | 9.2 | 10.7 | 9.18 | 9.73 | 9.22 | 9.83 |
| GRAVY | | -0.916 | -0.669 | -0.598 | -0.726 | -0.907 | -0.543 | -0.860 |

**Table S2. Comparison of physicochemical parameters of human secretory RNases.** pI and Grand average of hydropathy (GRAVY) were predicted using the ProtParam tool (<https://web.expasy.org/translate/>).
